# Who let the dogs out? Exploring the spatial ecology of free‐roaming domestic dogs in western Kenya

**DOI:** 10.1002/ece3.7317

**Published:** 2021-03-20

**Authors:** Patrick Muinde, Judy M. Bettridge, Filipe M. Sousa, Salome Dürr, Ian R. Dohoo, John Berezowski, Titus Mutwiri, Christian O. Odinga, Eric M. Fèvre, Laura C. Falzon

**Affiliations:** ^1^ International Livestock Research Institute Nairobi Kenya; ^2^ Institute of Infection, Veterinary, and Ecological Sciences University of Liverpool Liverpool UK; ^3^ Veterinary Public Health Institute University of Bern Bern Switzerland; ^4^ Atlantic Veterinary College University of Prince Edward Island Charlottetown Canada; ^5^ Jomo Kenyatta University of Agriculture and Technology Nairobi Kenya; ^6^ Present address: World Animal Protection Nairobi Kenya; ^7^ Present address: Natural Resources Institute University of Greenwich Chatham Maritime UK

**Keywords:** *Canis lupus familiaris*, Habitat Utilization, Home Range, Kenya, Roaming behavior, Utilization Distribution, Zoonoses

## Abstract

The spatial ecology of free‐roaming dogs determines their role in the transmission of zoonoses. This study describes the geographic range of and identifies sites frequently visited by free‐roaming domestic dogs in western Kenya.

Eight sites in Busia county, western Kenya, were selected. At each site, ten dog‐keeping households were recruited, a questionnaire was administered, and a GPS logger was fixed around the neck of one dog in each household. Loggers were programmed to capture the dog's position every minute, for five consecutive days. Individual summaries of GPS recordings were produced, and the daily distance traveled was calculated. 50% and 95% utilization distribution isopleths were produced, and the area within these isopleths was extracted to estimate the size of the core and extended Home Ranges (HRs), respectively. Linear regression analyses were performed to identify factors associated with the movement parameters. The centroid points of the 10, 50, and 90% isopleths were reproduced, and the corresponding sites identified on the ground.

Seventy‐three dogs were included in the final analyses. The median daily distance traveled was 13.5km, while the median core and extended HRs were 0.4 and 9.3 ha, respectively. Older dogs had a larger extended HR and traveled more daily, while the effect of sex on dog movement depended on their neutering status. Dogs spent most of their time at their household; other frequently visited sites included other household compounds, fields, and rubbish dumps. One of the centroids corresponded to a field located across the international Kenya–Uganda border, emphasizing the fluidity across the border in this ecosystem. Multiple dogs visited the same location, highlighting the heterogeneous contact networks between dogs, and between dogs and people.

The field data presented are of value both in understanding domestic dog ecology and resource utilization, and in contextualizing infectious and parasitic disease transmission models.

## Graphical abstract

In this study, we explored the spatial ecology of free‐roaming domestic dogs in western Kenya. High individual heterogenity occurred in dog movement patterns, and sex and neutering interplayed to shape dog movement patterns.

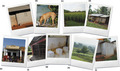

## 1 INTRODUCTION

Domestic dogs (*Canis lupus familiaris*) descended from gray wolves (*Canis lupus*) and were the first animals to be domesticated 12,000 to 16,000 years ago (Clutton‐Brock, 2017; Vonholdt & Driscoll, 2017). Since then, dogs have developed a complex relationship with humans and are present in every territory inhabited by man (Daniels & Bekoff, 1989; Hughes & MacDonald, 2013). Dogs fulfill various roles for humans, including as hunting aides, service and assistance dogs, and pets (Hart & Yamamoto, 2017; Serpell, 2017). Moreover, the physical and psychological benefits accrued through canine companionship are increasingly recognized (Gupta, 2017; Metsel, 2017; Mills & Hall, 2014; Westgarth et al., 2019). However, there is also a negative aspect to man's symbiotic relationship with dogs, as they may prey on or harass livestock, inflict injuries, and act as carriers of zoonotic diseases, including rabies (Knobel et al., 2005; Metsel, 2017).

These negative aspects are further exacerbated in free‐roaming dogs, which constitute around 75% of the global dog population (Hughes & MacDonald, 2013). Free‐roaming dogs may be owned or unowned, but their defining characteristic is that they are not effectively restrained but remain reliant on human communities for food and shelter (Boitani et al., 2017; Slater, 2001). This unrestricted movement places free‐roaming dogs in a unique position as they have access to their community spaces and surrounding natural habitat (Maher et al., 2019; Majumder et al., 2016), as well as occasionally being welcome inside households (Watson‐Jones & Macpherson, 1988). Free‐roaming dogs may therefore interfere with wildlife through predation, competition, or hybridization (Berman & Dunbar, 1983; Hughes et al., 2017). They may also cause public nuisance through excessive barking and soiling of community spaces, and their welfare is often compromised (Hiby & Hiby, 2017; Rubin & Beck, 1982). Furthermore, free‐roaming dogs’ uncontrolled movement increases their exposure to pathogens, thereby facilitating disease spread and posing a serious threat to human health (Garde et al., 2016; van Kesteren et al., 2013; Kwoba et al., 2019).

In Kenya, there are 5 to 6 million dogs, of which more than 80% are thought to be owned (ZDU, 2017). However, it is common practice to allow owned dogs to roam freely, as shown in several studies conducted in various Kenyan counties (Kitala et al., 2001; ZDU, 2014; Kwoba et al., 2019). Moreover, the dog population is on the rise (Kitala et al., 2001), as is seemingly the incidence of dog bites (Mbenywe, 2018). This is of particular concern since a number of dog‐borne zoonoses are known to be present in the country, including rabies (Bitek et al., 2019). Indeed, rabies is ranked among the top five priority diseases in Kenya (Munyua et al., 2016), and in 2014, the Zoonotic Disease Unit launched its National Strategy with the goal of eliminating dog‐mediated human rabies by 2030. This strategy highlights the need for studies about dog demography, behavior, and spatial ecology, as these are crucial for guiding and assessing disease control management strategies (ZDU, 2014; ZDU, 2017).

Indeed, transmission of zoonotic diseases depends on proximity between hosts, which in turn is driven by their movement and interactions (Floyd et al., 2019; Parsons et al., 2014). Understanding dogs’ spatial ecology will therefore provide insight on where animals may be at risk of exposure, pinpoint hotspots of disease transmission, and help optimize population control and intervention strategies (Hudson et al., 2019; van Kesteren et al., 2013).

An important aspect of animal movement, which provides insight into an animal's behavior, is its Home Range (HR). This is the area an animal commonly traverses to pursue daily activities such as feeding, mating, and caring for young (Burt, 1943; Powell & Mitchell, 2012; Worton, 1987). The HR represents how the animal understands and uses its environment, hence revealing aspects of the animal's familiarity with, and utility of, an area (Powell, 2000; Powell & Mitchell, 2012). The HR is influenced by both intrinsic and extrinsic factors, which in turn may determine the animal's range of movement (Boitani et al., 2017; Burt, 1943; Croft et al., 2008; Dürr et al., 2017; Pérez et al., 2018).

While the HR shows the range of the animal's movement, it is also important to understand the interior of that range, and how it is used by the animal (Powell, 2000). The Utilization Distribution (UD) is a common procedure to assess the use of space within a HR by integrating the time the animal spent in a specific area throughout the duration of the study (Benhamou & Riotte‐Lambert, 2012). The procedure uses a probability density function to look at the intensity of use of the various areas, which is then expressed as the probability of an animal being within any part of its HR (Powell, 2000; Worton, 1987). The UD therefore provides information on both the spatial and temporal use of an area, thus highlighting risk areas for potential disease transmission and intensity of environmental contact (Hudson et al., 2019).

In this study, we aimed to understand the spatial ecology of free‐roaming domestic dogs in western Kenya, both in terms of the area they regularly traverse, and the sites they commonly visit. Specifically, the objectives of this study were as follows: i) to describe dog demography and management practices; ii) to describe the HR of free‐roaming dogs and factors that may influence it; and iii) to identify sites located within the dogs’ UD areas. Our study site is in western Kenya, broadly representative of the Lake Victoria Crescent ecosystem, the area of East Africa with the highest domestic animal and human population densities and with rapidly evolving farming systems (Falzon et al., 2019; Fèvre et al., 2017).

## 2 MATERIALS AND METHODS

### 2.1 Study area

**FIGURE 1:**
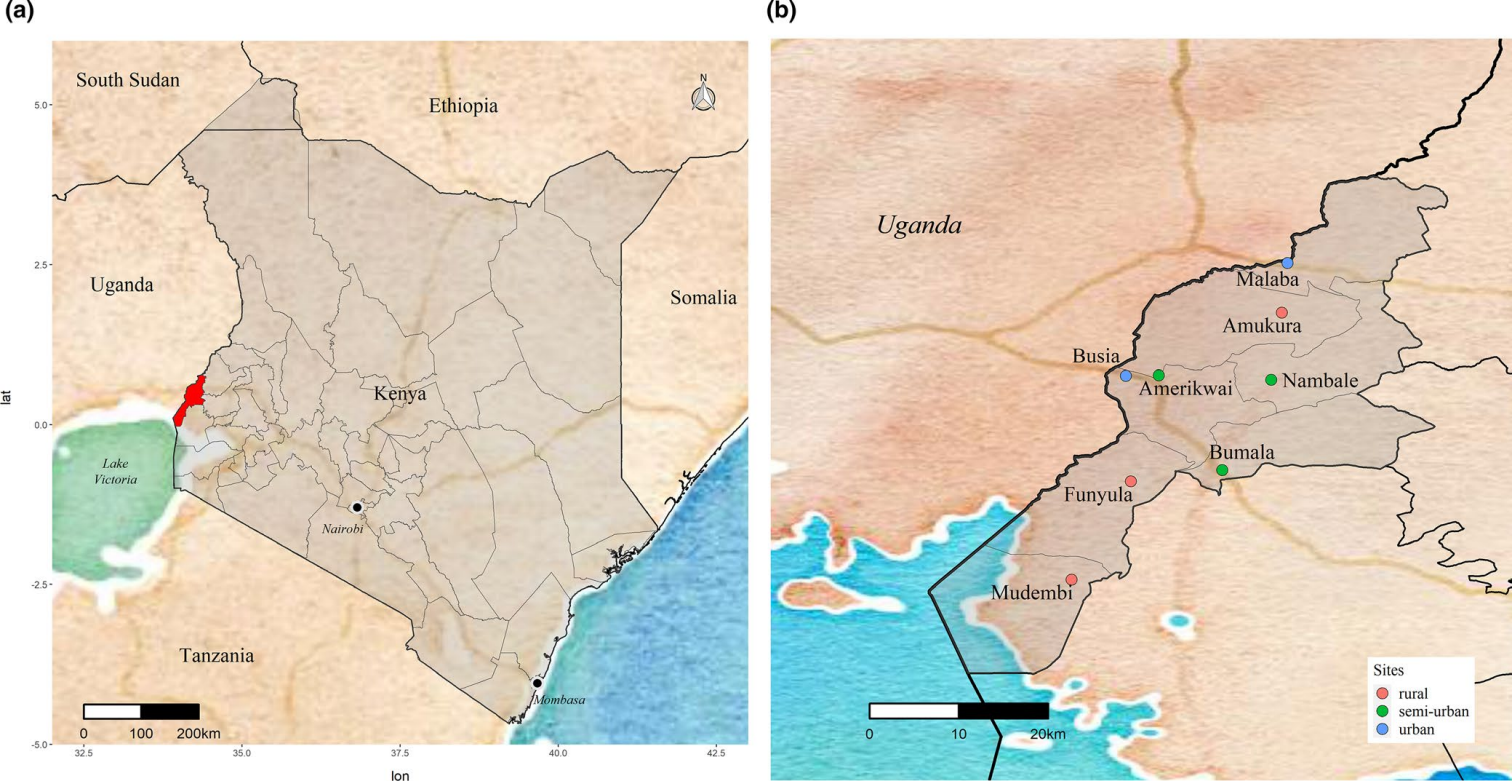
A map of Kenya, highlighting the location of Busia county (a), and a map indicating the location of the eight sites within Busia county (b)

The study population was free‐roaming owned dogs in Busia county, western Kenya. This county borders Uganda to the west (Figure 1a), and has a tropical and humid climate, with an average annual temperature of 25°C and an annual mean precipitation ranging between 900 and 1,500 mm.

Eight sites in Busia county were selected to capture the diversity of the study area. These sites included two urban border towns [Busia town and Malaba], three urban/peri‐urban centers [Amerikwai, Bumala, and Nambale], and three rural areas [Amukura, Funyula, and Mudembi] (Figure 1b). Data were collected during two periods: May‐June 2017 and June‐July 2019.

### 2.2 Household and dog selection

Our inclusion criteria were households that owned free‐roaming dogs, where free‐roaming was defined as the dog being allowed to roam off‐leash or free from kennel for any amount of time during the day and/or night. Households that owned dogs younger than 6 months or dogs that appeared unhealthy were not included.

At each site, we liaised with a local community leader to help us identify ten households that met our inclusion criteria. At each household, the rationale of the study was explained and written consent was obtained. The household GPS coordinates were recorded, and an electronic questionnaire was administered by two of the co‐authors (PM and TM) who were trained in questionnaire administration and knowledgeable in the subject. The questionnaire was designed in English (S1) but was interpreted into Kiswahili upon administration by the two interviewers who also answered any follow‐up questions on the study.

**FIGURE 2:**
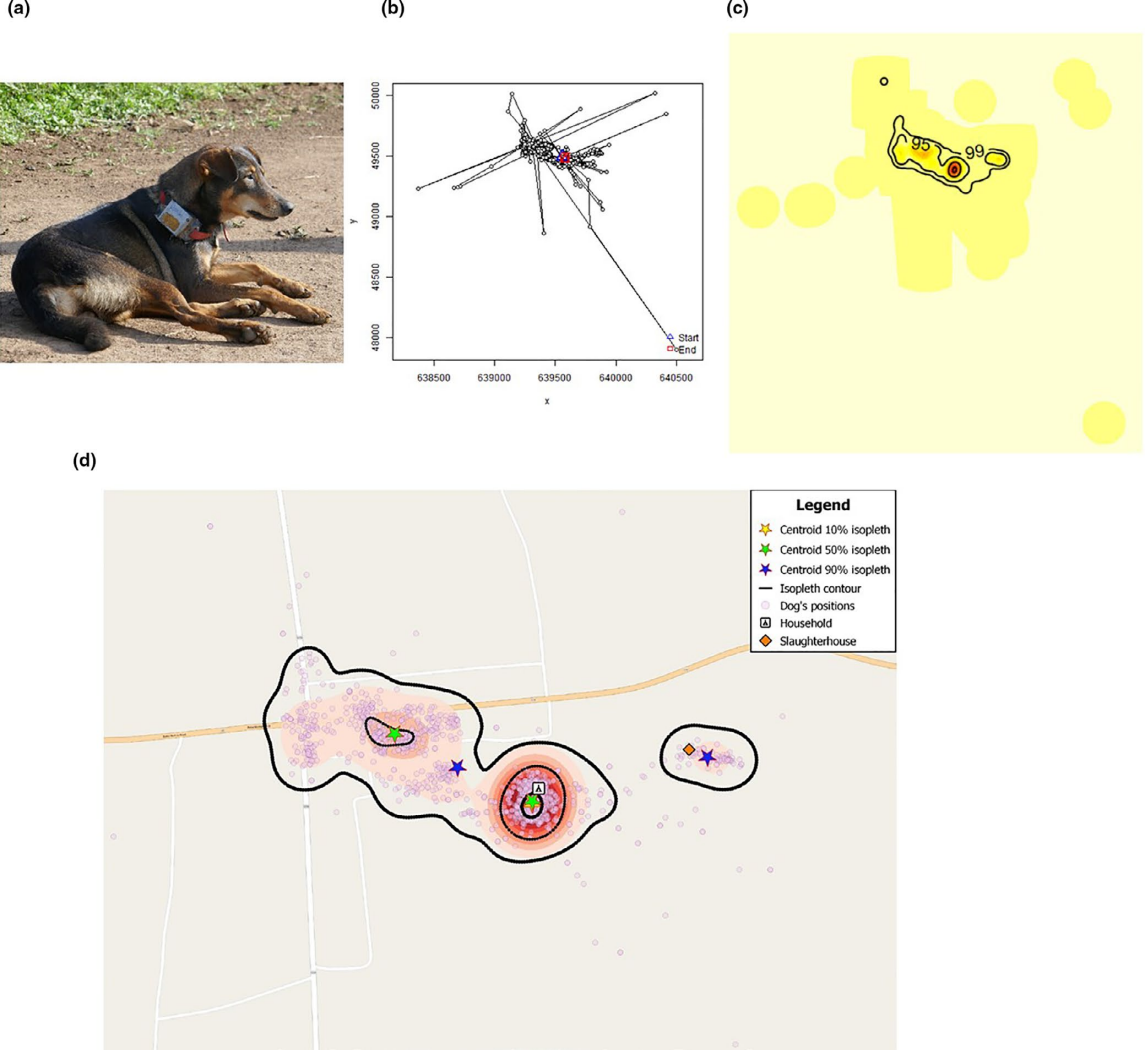
A photograph of one of the dogs that was tagged in the study, illustrating the placement of the GPS logger (a); the trajectory of the GPS coordinates recorded for dog 4288 (b); the utilization distribution map used to determine the core (50%) and extended (95%) home range of dog 4288 (c) and the utilization distribution map used to define the 10%, 50% and 90% isopleths, and their centroids, for dog 4288 (d)

Only one dog per household was included in the study. If more than one dog was present, we selected the dog based on the owner's recommendations and ease of capture. We also attempted to include an equal number of male and female dogs at each site. The selected dog was manually restrained and a Mobile Action i‐gotU GT‐600 (Mobile Action Technology, Inc., Taiwan) GPS logger was strapped around its neck (Figure 2a). The GPS loggers were set to record location data every minute whenever the dog was in motion, and for a period of five days, after which the collars were retrieved. The time and date when the collar was put on the neck of the dog, and when it was later retrieved, were recorded.

### 2.3 Data cleaning and analysis

#### 2.3.1 GPS data

The GPS data collected by the loggers were downloaded using the free *@trip pc* (http://www.atrip.com) software and then exported as an individual.csv file for each dog into R statistical software (https://www.R‐project.org, version 3.4.0) (R Core Team, 2017) for further data cleaning and analysis.

The GPS data were cleaned and analyzed as described in a prior study (Dürr & Ward, 2014). The GPS data coordinates were projected into Universal Transverse Mercator WGS 36 N, and dogs that had less than 24 hr of GPS data (*n* = 1) were excluded. The average speed of travel between every two fixes was estimated, and fixes with a speed greater than 20km/h were excluded, based on visual inspection of speed histograms and the assumption that community dogs are unlikely to sustain such a speed for an entire minute (Dürr & Ward, 2014; Hudson et al., 2017; Molloy et al., 2017).

Individual summaries of GPS fixes were produced for each dog and used to estimate both the daily and total mean distance traveled (Figure 2b). For each dog, the HR was estimated with the “*adehabitatHR”* package in R (Calenge, 2006) using the biased random bridges method to determine the UD. This is a movement kernel‐based technique that links two successive fixes, and then interpolates between them, to give a more realistic animal movement pattern (Benhamou, 2011). The 50% and 95% isopleths were produced, and the area within these isopleths was extracted to estimate the size of the core and extended HRs, respectively (Figure 2c). To determine the proportion of time each dog spent within their respective household, a standardized area with a 20m radius around each household was defined as the household area. This measurement was decided upon following visual inspection of participating households, and the measurement of some of the households included in the study. The time spent within this area was then estimated as a proportion of the overall recording period.

#### 2.3.2 Risk factor analysis

**FIGURE 3:**
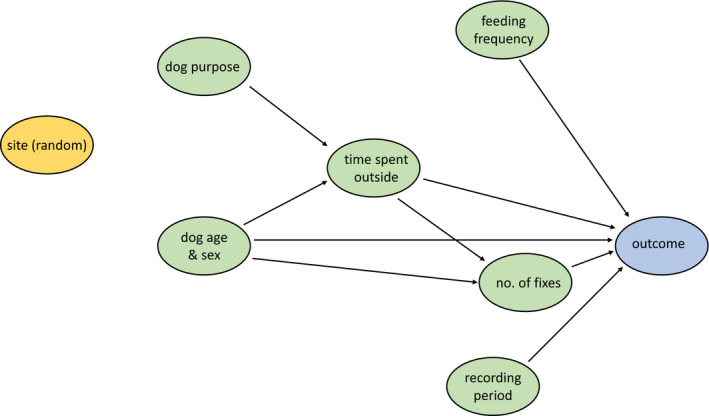
A causal diagram to identify putative relationships between exposure variables of interest and movement outcomes of 73 free‐roaming dogs in western Kenya

Risk factor analysis for the four outcomes of interest, namely daily distance traveled (km), core and extended HRs (hectares), and proportion of recording time spent within the household (%), was conducted using Stata Statistical Software: Release 14 (College Station, TX: StataCorp LP). A causal diagram was developed to identify putative relationships between exposure variables of interest and outcomes, and to guide the modeling process (Figure 3). The four outcome variables were checked for collinearity and for normal distribution; variables that were not normally distributed were transformed as needed. Subsequently, mixed linear regression models were developed for each of the four outcome variables, with site included as a random effect to account for spatial clustering within each site. Outliers and influential observations were assessed, and their effect on the final model was recorded. Given the limited sample size, statistical significance was considered when the p‐value was ≤ 0.1 to reduce the probability of a Type 2 error, and the intracluster coefficient was computed as the proportion of overall variation due to variation between groups.

#### 2.3.3 Identification of sites within area used by dogs

To identify sites where dogs spent more of their time, the 10% and 50% UD isopleths (representing the 10% and 50% probability of relocating the animal within that area) were reproduced. Additionally, the 90% UD isopleths were also reproduced so as to explore other sites present within the areas used by the dogs. The centroid point of each isopleth area was then estimated as the arithmetic mean value of the corresponding area, based on the latitudes and longitudes. Depending on the shape and size of the isopleth, dogs could have one or more centroids per isopleth (one centroid for each polygon within the isopleth) (Figure 2d). We then went back to the eight sites, entered the GPS coordinates of each centroid point into Google Maps, and walked to the site that corresponded to the centroid. Once at the site, we noted the location and features of the site, and if the dog owners or other persons were present, we enquired whether they observed dogs at these sites.

### 2.4 Ethical approval

Ethical approval for this study was granted by the International Livestock Research Institute Institutional Animal Care and Use Committee (Reference number 2017–10) and by the Social Science Research Ethical Review Board at the Royal Veterinary College (Reference number URN SR2017‐1084).

## 3 RESULTS

Eighty dogs were recruited (ten at each site) for the study; however, seven were excluded from further analysis because the data‐loggers could not be retrieved (*n* = 4), they had recorded for < 24 hr (*n* = 1), or the questionnaire data were not available (*n* = 1). We also realized that two dogs that were sampled from one site belonged to the same household, and the one that had least data was therefore excluded from further analysis.

### 3.1 Dog demographic and movement characteristics

The mean number of people living in each household was 8.6 (median = 7; range = 2–23), while the mean number of dogs in each household was 2.1 (median = 1; range = 1–10). Of the 73 dogs included in the study, the majority were between 1 and 5 years old (*n* = 56; 77%). Forty dogs were female, of which 5 were spayed, while 10 of the 33 male dogs were castrated. Almost all the dogs included in the study (*n* = 70; 95.9%) were kept for security purposes.

While all dogs were allowed to roam freely for part of the day or night, more than half of the owners reported that, on a typical day, their dogs roamed for less than 2 hr (*n* = 46; 64%). Most owners also reported that they fed their dogs daily (*n* = 71; 97%). More than half of the owners said that their dogs had neither been dewormed (*n* = 45; 63%), nor vaccinated against rabies (*n* = 44; 60%) in the last 12 months.

The recording period ranged between 25 and 152 hr; however, the majority of the loggers (*n* = 63; 86%) recorded for at least 96 hr (i.e., 4 days), and the median recording period was 123 hr (i.e., 5.1 days). After data cleaning, the collars had recorded a median number of 21 fixes per hour per dog (ranging between 2 and 33 fixes per hour).

Dogs traveled a median of 13.5 km daily, though this ranged between 2.0 and 24.5km. Both the HR area and shape varied considerably between dogs (S2), and across the different study sites (Table 1). The core HR (50% isopleth) ranged between 0.4^10–5^ and 2.4 ha (0.04 to 24,000 m^2^), with a median of 0.4 ha (interquartile range = 0.26–0.60 ha), while the extended HR (95% isopleth) ranged between 1.2 and 97.4 ha, with a median of 9.3 ha (interquartile range = 4.64–16.93 ha). The dogs spent a median of 31.9% (range = 0.2 to 79.4%) of the recording period within the household compound.

**TABLE 1:** The number of dogs, and their movement parameters, included in eight sites in Busia County

Study site	Type	No. of dogs	Median daily distance traveled (km)	Median core Home Range (ha)	Median extended Home Range (ha)	Median time spent in household (%)
Amerikwai	Peri‐urban center	9	12.88	0.28	4.09	45.69
Amukura	Peri‐urban center surrounded by rural areas	9	14.83	0.50	14.29	28.69
Bumala	Urban center with peri‐urban and rural areas	10	13.85	0.59	7.63	29.64
Busia	Border town	9	11.06	0.25	5.42	47.82
Funyula	Peri‐urban center surrounded by rural areas	10	13.32	0.51	11.36	39.30
Malaba	Border town	8	13.27	0.63	10.41	26.82
Mudembi	Rural area	10	16.03	0.49	11.38	33.94
Nambale	Urban center with peri‐urban and rural areas	8	15.22	0.49	13.14	13.72
Overall		73	13.47	0.39	9.3	31.91

### 3.2 Regression analysis

Of the seven explanatory variables identified in the causal diagram (Figure 3), two (i.e., dog purpose and frequency of feeding) were excluded from further analysis due to lack of variation. The remaining five variables (dog age, dog sex, time spent outside household, number of fixes, and recording period) were included for further analysis.

The four outcome variables were not highly correlated (Pearson's correlation coefficient < 0.7) and were therefore retained as separate outcomes. Both the core and extended HR were not normally distributed and were transformed using a square‐root and logarithmic transformation, respectively.

The results of the unconditional associations between the four outcome variables and the explanatory variables are presented in Table 2, while the results of the final mixed linear regression models are presented in Table 3. As there was no evidence of difference between the 1–5 years and > 5 years age categories in any of the multivariable models, the age variable was dichotomized, that is, <1 year and ≥ 1 year old. The number of fixes per hour was positively associated with the daily distance traveled (coefficient = 0.40) and the proportion of time spent within the household (coefficient = 0.86), and negatively associated with the core HR (coefficient=−0.00009) (Table 2). However, following removal of 11 outliers (i.e., 8 observations that had < 10 fixes/hour and 3 observations that had > 30 fixes/hour), the variable was no longer statistically associated with the core HR and proportion of time spent within the household. Nonetheless, we retained the full variable in the final model as we could not find any justification for removing these 11 outliers. Age was positively associated with the daily distance traveled and the size of the extended HR, and negatively associated with the percentage of time spent in the household area. Sex was marginally associated with the daily distance traveled, whereby the effect of dog's sex on the distance traveled depended on their reproductive status. Specifically, castrated males tended to travel less, compared to intact males, while the inverse was observed in females whereby neutered females tended to travel more, compared to intact females. Finally, the variables sex and age were retained in all the final models, regardless of their p‐values, since they were identified as confounders based on our causal diagram.

**TABLE 2:** Unconditional associations between the four outcome variables and the explanatory variables investigated for their putative association with various dog movement parameters obtained from 73 free‐roaming dogs in western Kenya

			Outcome variables			
Explanatory variables	Descriptive statistics	*N* (%)	Mean (*SD*) & median daily distance traveled (km)	Mean (*SD*) & median core Home Range (ha)	Mean (*SD*) & median extended Home Range (ha)	Mean (*SD*) & median time spent in household (%)
Dog age	<1 year	5 (7)	11.96 (3.38) 14.0	0.34 (0.16) 0.28	5.96 (4.02) 5.53	39.98 (16.24) 38.16
	1–5 years	56 (77)	13.79 (4.46) 13.50	0.54 (0.43) 0.38	13.90 (16.13) 9.33	31.68 (18.15) 30.02
	>5 years	12 (16)	12.68 (4.80) 12.60	0.59 (0.43) 0.51	14.69 (10.84) 14.82	28.37 (18.17) 30.75
			*p* =.49	*p* =.49	*p* =.20	*p* =.25
Dog sex	Intact male	23 (31)	14.28 (3.56) 14.0	0.52 (0.47) 0.36	14.52 (15.64) 9.36	32.74 (18.30) 35.68
	Castrated male	10 (14)	11.63 (4.57) 10.13	0.30 (0.19) 0.25	9.97 (9.13) 6.29	38.47 (15.87) 44.36
	Intact female	35 (48)	13.23 (4.88) 13.09	0.59 (0.36) 0.57	11.49 (8.32) 9.30	30.56 (18.47) 29.87
	Spayed female	5 (7)	15.20 (4.35) 15.03	0.71 (0.77) 0.36	29.70 (38.27) 14.81	21.37 (15.79) 18.08
			*p* =.31	*p* =.05**	*p* =.29	*p* =.32
Time spent outside the household	<2 hr	46 (64)	13.85 (4.98) 14.24	0.55 (0.46) 0.41	14.83 (17.54) 9.18	30.59 (18.10) 29.56
	2–6 hr	17 (24)	12.40 (3.57) 12.88	0.41 (0.20) 0.33	9.29 (6.35) 8.67	36.16 (19.05) 40.86
	>6 hr	9 (12)	13.86 (2.86) 13.06	0.71 (0.49) 0.52	14.95 (11.33) 9.67	29.15 (16.86) 35.22
			*p* =.49	*p* =.22	*p* =.56	*p* =.58
Number of fixes/hour	Median (Range)	20.6 (1.84–33.47)	*p* <.001***	*p* =.02**	*p* =.44	*p* =.002***
Number of fixes/hour†	Median (Range)	20.89 (11.40–29.90)	*p* <.001***	*p* =.73	*p* =.28	*p* =.84
Hours of recording	Median (Range)	122.57 (25.35–151.61)	*p* =.73	*p* =.46	*p* =.85	*p* =.82

^†^ Removal of 11 outliers with values < 10 or > 30 fixes/hour.

* p‐value ≤ 0.1; **p‐value ≤ 0.05; ***p‐value ≤ 0.001.

**TABLE 3:** Final mixed linear regression models for four outcome variables related to movement of 73 free‐roaming dogs in western Kenya. No variables were significant for the core home range so no model is presented

	Coefficient	SE†	p‐value	Intracluster coefficient
**Daily distance traveled**				0.00078
Dog age	3.66	1.56	0.02**	
Dog sex			0.10*	
Castrated male	−2.63	1.25		
Intact female	−1.20	0.89		
Spayed female	0.90	1.61		
Number of fixes/hour	0.41	0.06	<0.001***	
**Extended Home Range**				0.14
Dog age	5.37	2.40	0.05**	
Dog sex			0.21	
Castrated male	0.32	2.04		
Intact female	0.66	1.66		
Spayed female	2.15	2.45		
**Percentage time spent in household**				0.2
Dog age	−12.84	7.51	0.09*	
Dog sex			0.20	
Castrated male	8.66	6.30		
Intact female	−0.71	4.29		
Spayed female	−8.50	7.84		
Number of fixes/hour	0.88	0.27	0.001***	

^†^ SE = Standard Error

* p‐value ≤ 0.1; **p‐value ≤ 0.05; ***p‐value ≤ 0.001

### 3.3 Sites within area used by dogs

**FIGURE 4:**
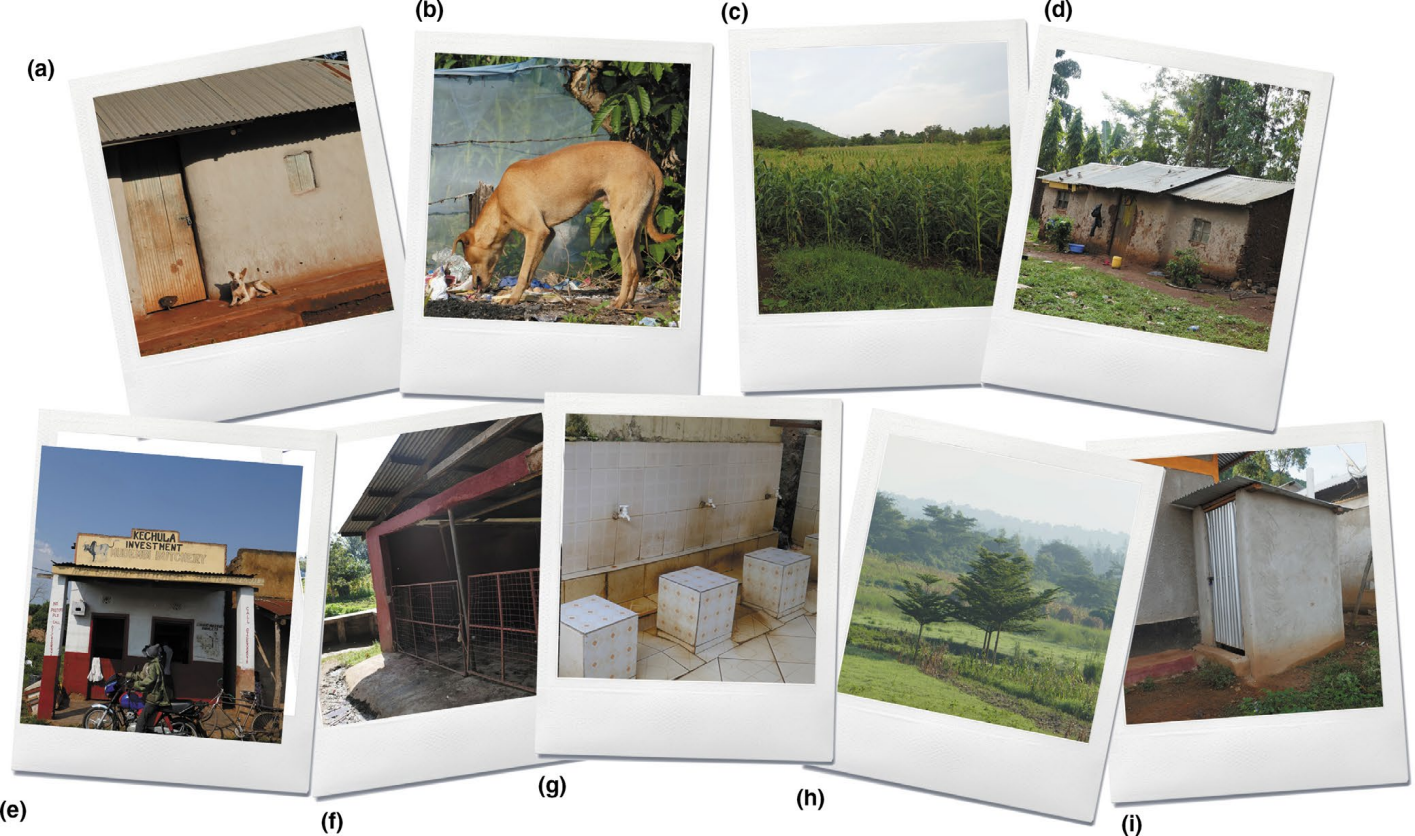
Sites frequently visited by 73 free‐roaming owned dogs in western Kenya included their own household (a), rubbish dumps (b), fields (c), and other household compounds (d). Other sites present within the area used by the dogs included a butchery (e), a slaughterhouse (f), water taps in a mosque (g), a field in Uganda (h), and a pit latrine (i)

The median number of 10% and 50% centroids per dog was 1, and the median area (and range) of the corresponding polygon areas was 841.24m^2^ (0.5–3683.97m^2^) and 7,189.74m^2^ (326.36–37,849.84m^2^), respectively. The majority of both the 10 and 50% isopleths corresponded to the dog's household (Table 4 and Figure 4). Other preferentially visited sites included other household compounds, fields, and rubbish dumps.

**TABLE 4:** A summary of the locations corresponding to the centroid points for the 10%, 50%, and 90% utilization distribution isopleths of 73 dogs tagged in 8 sites in Busia County

Location of centroid point	10% isopleth centroids	50% isopleth centroids	90% isopleth centroids
	*N* = 79	*N* = 96	*N* = 208
Dog's household	84.8%	68.8%	20.2%
Other household compound	7.6%	13.5%	24.0%
Field	5.1%	6.3%	24.0%
Rubbish dump	2.5%	8.3%	17.8%
Grassy area		1%	2.4%
Pit latrine		1%	0.5%
Butchery		1%	
Forest/bush area			2.9%
Roadside			2.4%
Slaughterhouse			1.9%
Water point			1.4%
Market space			1.0%
Church			0.5%
Holding ground			0.5%
Sand harvesting point			0.5%

The median number of 90% centroids per dog was 2, ranging between 1 and 19, and the median area (and range) of the polygons corresponding to these centroids was 18,420.04 m^2^ (25.63–485,979.10 m^2^). Noteworthy is that one of these sites corresponded to a field that was located across the international Kenya–Uganda border (dog 2770 [S3]), emphasizing the fluidity across the border in this ecosystem. Furthermore, in a number of study sites we observed that multiple dogs visited the same location (e.g., in Nambale, both dogs 2795 and 3753 visited the same rubbish dump), or one dog visited the household of another dog (e.g., in Mudembi, dog 2760 visited the household of dog 2747) (S3), emphasizing the heterogeneous contact networks that likely exist between dogs, and between dogs and people.

## 4 DISCUSSION

In this study, we described the spatial ecology of 73 free‐roaming dogs and identified sites which could act as hotspots for disease transmission. To our knowledge, this is the first study to collect field data on the extent and range of domestic dog movement in Kenya that can be used to inform and contextualize disease transmission models.

Most of the 73 dogs included in the study were adults, with fewer juvenile and older dogs. It is not surprising that there were fewer older dogs given that free‐roaming dogs are often left to fend for themselves, and therefore have a reduced survival rate (Daniels & Bekoff, 1989; MacDonald & Carr, 2017). Indeed, a study in Machakos county, Kenya, found that free‐roaming juvenile dogs had a survival rate of 50%, while their median life expectancy ranged between 2.8 and 2.9 years (Kitala et al., 2001). Therefore, few free‐roaming dogs live to become adults, resulting in high replacement rates in these populations. Thus, at any point in time, the population will have a significant proportion of dogs that are young and immunologically naïve to many zoonoses, further increasing the community risk of exposure to such diseases.

Almost all dogs included in this study were kept for security purposes, as also reported in other studies conducted in Kenya (Kitala et al., 2001; Kwoba et al., 2019), Nigeria (Mshelbwala et al., 2018), Zimbabwe (Butler & Bingham, 2000), Chile (Acosta‐Jamett et al., 2010), and rural areas of Bhutan (Rinzin et al. 2016). This reflects the utilitarian role of dogs in these areas, where economic and pragmatic considerations often influence the decision to acquire and keep a dog (Knobel et al., 2008; Serpell, 2004).

More than half of the respondents reported that, on a typical day, their dogs roamed for less than two hours. While in some cases this was based on the fact that the dog was confined for certain periods of the day, in many other cases the response was based on the owner's observations, who was not necessarily familiar with their dog's whereabouts. In fact, contrary to what we hypothesized in our causal diagram (Figure 3), we did not observe any significant association between the explanatory variable “time spent outside the household” and any of the movement parameters (Table 2). This suggests that the response to this question was not necessarily reflective of the dog's true movements.

In this study, 40% of the dogs had been dewormed or vaccinated against rabies in the last 12 months. While this is higher compared to the vaccination rates of 5% (Kwoba et al., 2019), 29% (Kitala et al., 2001), and 35% (Mucheru et al., 2014) reported in other Kenyan counties, it is still well below the recommended vaccination coverage of 70% required to break endemic cycles of rabies transmission (Coleman & Dye, 1996; WHO, 2020). Furthermore, Kitala et al. (2002) found that low vaccination rates may be counterproductive as they increase the stability of the rabies virus within the dog population, therefore allowing for endemic establishment of the virus. These findings highlight the need to increase awareness of responsible dog ownership, particularly among school children who are often those closely involved in dog care (Hiby & Hiby, 2017; Kitala et al., 2001).

This study focused on free‐roaming domestic dogs, which is the primary means of dog management in the study area, indeed in much of sub‐Saharan Africa. The primary reasons cited for allowing dogs to roam freely included to reduce the dogs’ aggressive behavior and because the dogs dislike being chained (Mutwiri, unpublished data). The field data obtained in this study indicate that the dogs moved considerably during the observation period, with some dogs traveling up to 24km daily, though heterogeneity between individual dogs was noted. The median core HR of 0.4ha reported in this study was similar to that described in other studies that also used the biased random bridge method (Dürr et al., 2017; Hudson et al., 2017; Molloy et al., 2017), while the median extended HR of 9.3 ha was slightly higher. However, as also described elsewhere (Dürr et al., 2017; Dürr & Ward, 2014; Hudson et al., 2017; Molloy et al., 2017), in our study we observed a wide range in both the core and extended HR of dogs, suggesting that heterogeneity in dog movement patterns is also present in western Kenya. Indeed, differences in the size and shape of the HRs were observed both between, and within, study sites. One putative reason for these differences may be the topography of the area, with major roads in the urban centers curtailing some of the dog's movements, compared to those dogs that lived further away from the urban centers. Another explanation could be related to management practices, such as frequency of feeding and dog purpose. Unfortunately, we were unable to explore the effect of these explanatory variables in our study due to lack of variation. Lastly, we think that “human geography” may also play a role, whereby the dog's behavior is highly connected with the way people move and behave in different places, consequently influencing the shape and size of their HRs.

In our study, older animals (>1 year) had an extended HR that was 5.40 ha larger, and traveled 3.66km more daily, compared to younger animals. Pal et al. (1998) also found that adult dogs had larger HRs, compared to younger dogs. Dogs usually establish a HR when they become adults (Burt, 1943), and adult dogs tend to dominate over subadult and juvenile dogs (Cafazzo et al., 2010). This may therefore explain the larger range of movement observed in the adult dogs included in this study. However, it must be noted that the effect of age on both distance traveled and time spent in the homestead was mediated through the number of fixes, whereby young dogs had more fixes recorded per hour, which therefore made it look like there was an overall effect of age on distance. Furthermore, the effect of age should be treated with caution as there were only 5 dogs in the referent group (i.e., <1 year).

In this study, sex was unconditionally associated with both the core HR and the daily distance traveled. Specifically, castrated males tended to travel less, compared to intact males, while the inverse was observed in females whereby neutered females tended to travel more, compared to intact females. Different effects of sex and neutering on dog roaming behavior are reported in the literature. While Daniels (1983), van Kesteren et al. (2013), and Maher et al. (2019) found no effect of sex on dogs’ movement patterns, Vaniscotte et al. (2011) and Boitani et al. (2017) found that male dogs tended to travel more than females, possibly because of their increased territorial behavior. Similarly, while neither Garde et al. (2016) nor Maher et al. (2019) found an effect of neutering on dog movement, Hopkins et al. (1976) conclude that roaming behavior was reduced by 90% following castration, and Molloy et al. (2017) found that neutered dogs had smaller core HRs compared to intact dogs. Our findings, similar to those reported by Dürr et al. (2017), suggest that the effect of neutering on dog movement may vary depending on the dog's sex, whereby intact males traveled more, while intact females traveled less, compared to their neutered counterparts. However, it must be noted that the variable sex was not significant overall, possibly due to small sample size, and further research is required before drawing any conclusions.

The extended HR and proportion of time spent in the household varied considerably between study sites, and the final model intracluster coefficients showed that 14% and 20% of the variation in these outcomes, respectively, was due to variation between the sites (Table 3). Rinzin et al. (2016) reported that dogs in rural areas are more likely to roam, compared to dogs in urban areas. In this study, we were unable to further explore the impact of rural versus urban settings on the dogs’ movement due to the risk of misclassification bias, whereby the classification of the study site did not always represent the location of the household within that study site. Furthermore, our sample size was insufficient to do separate analyses by setting. However, we recognize that factors at the community level might also influence the dogs’ spatial ecology and therefore merit future research attention.

Dogs spent a median of 32% of the study duration within their household, and this corroborates with the finding that the majority of the 10% and 50% centroid points also corresponded to the dogs’ household. This is similar to the observation made in other studies that free‐roaming dogs tend to stay close to their homes (Berman & Dunbar, 1983; Daniels, 1983; Dürr et al., 2017; van Kesteren et al., 2013; Pérez et al., 2018; Vaniscotte et al., 2011). It must be noted that almost all dogs included in this study were kept for security purposes, which may further explain their observed site fidelity. Other frequently visited sites included other household compounds, crop fields, and rubbish dumps. Free‐roaming dogs tend to choose sites with increased human activity, such as human compounds, as they provide an opportunity to beg for food (Majumder et al., 2016). Similarly, dogs are known to commonly visit crop fields (Parsons et al., 2014), and in this study area, dogs are often kept in fields to protect crops from wildlife, particularly from primates which are well known for raiding crops such as maize and bananas (Naughton‐Treves et al., 1998).

Rubbish dumps provide a locally abundant food resource for free‐roaming dogs (Daniels & Bekoff, 1989; Mshelbwala et al., 2018; Serpell, 2017), and several authors have observed dogs scavenging on domestic waste (Beck, 1973; Boitani et al., 2017; Bombara et al., 2017; Kitala et al., 2001; Kwoba et al., 2019; MacDonald & Carr, 2017; Mangalam & Singh, 2013). It is therefore not surprising that dogs included in this study were familiar with and frequently visited rubbish dumps in the area. Olugasa et al. (2014) found a strong correlation between access to food sources and spatial distribution of animal bite injuries to humans in south‐eastern Nigeria, highlighting how such sites may pose a threat to public health if not properly managed. Indeed, a study in Aragón, Spain, observed a decrease in the stray dog population following improved waste management (Sobrino et al., 2009). Controlling dog's access to human food waste is therefore an important facet of dog management to improve public health (Hughes & MacDonald, 2013; ZDU, 2014; Hughes et al., 2017).

Other sites present within the areas used by the dogs included water sources, grazing areas, pit latrines, and slaughterhouses. Some of these sites are important from an ecological and public health perspective as they suggest the possibility for dogs to be involved in the transmission and propagation of a number of diseases. Also noteworthy was the observation that one of the dogs traveled to neighboring Uganda during the study. Animal movement can result in the introduction of transboundary animal diseases (Fèvre et al., 2006), and international cross‐border collaborations are therefore essential to manage and reduce the risk of disease import. Finally, we also observed that a few dogs visited the same sites or visited each other's household. This creates an opportunity for dogs to interact, therefore facilitating the dissemination of diseases such as rabies. Future research should investigate the social network of these dogs to better elucidate dog to dog interactions, and how these may impact the transmission of pathogens.

The number of fixes recorded was significantly associated with the daily distance traveled. For every unit increase in the number of fixes recorded, the daily distance traveled increased by 0.41 km. The number of fixes recorded was also significantly associated with the percentage of time spent in the household, though this effect disappeared once the 11 outliers were removed. The GPS loggers used in the study were set to record a fix every minute. However, the median number of fixes recorded per hour was 21, indicating that a fix was recorded approximately every 3 minutes, and this variation in number of fixes recorded impacted the other movement parameters. In this study we chose to use the “movement enabled” function of the GPS logger, whereby the loggers would only record a fix when the dog was in motion, to save on battery life. However, a pilot study conducted by some of the authors found that the logger recorded a fix following the slightest motion. This suggests that, based on this function alone, the logger would only not have recorded when the dog was perfectly still. It is therefore likely that other reasons also contributed to the fewer number of fixes recorded in this study. To calculate a fix, the GPS antenna triangulates signals from three or more satellites within a short period of time. Factors that influence satellite accessibility, including GPS collar orientation and habitat features such as tree height and canopy closure, excess rainfall, and atmospheric conditions (Dussault et al., 1999; Parsons et al., 2014) may have resulted in fewer signals.

In this study, erroneous locations in the dataset were screened for by setting a specific threshold for speed between two locations. We recognize that further filtering steps to control for locational error in our dataset, including using measures of estimated horizontal position error or dilution of precision, or specifying further thresholds for distance or turning angles between locations, might have removed some additional locations with high error (Morris & Conner, 2017).

The short duration of the recording period in this study precluded us from looking into the effect of extrinsic factors, such as season, on movement patterns. Several studies have identified temporal variation in dogs’ peak activity (Berman & Dunbar, 1983; Boitani et al., 2017; van Bommel & Johnson, 2014; Maher et al., 2019; Pérez et al., 2018; Rubin & Beck, 1982), possibly linked to human activity, environmental conditions, or innate behavioral traits. The temporal variation in dogs’ movement patterns therefore merits future research attention, as this would shed light on when dogs visit and congregate at specific sites, which in turn would have critical implications for zoonotic disease control, dog population management, and owner education.

We are aware that the isopleth centroid points, which represent the arithmetic mean value for a two‐dimensional distribution, are a theoretical concept and do not correspond to a real point. They may therefore not always be representative, particularly for some of the polygons with wider areas within the 90% isopleths. Furthermore, they do not allow for inferences on whether the animal was directly drawn to a specific site or whether its movement there was driven by a purpose. We therefore only describe these as sites present within the area used by the dog. The fact that many centroids matched the dog's household validated the link between the data and the real world, and when possible, we enquired with any persons present at the time of the visit to further confirm whether dogs frequented that site. We note though that we could not find reports of any methods to identify within‐animal clustering of domestic animals using GPS points collected in a real‐world experiment. Future research efforts on the use of time series for exploring domestic animal ecology and behavior could help address this gap in methodologies.

## 5 CONCLUSION

This study provides field data on the range of distance traveled and area covered by dogs, while shedding light on the heterogeneity between individual dog movement patterns. This information can be used to ensure that disease transmission models investigating the impact of intervention strategies or vaccination coverage rates are tailored for the local Kenyan situation. We also highlight how dogs regularly visit rubbish dumps, and the potential for them to access other sites of public health significance or cross international borders. Better waste management strategies and regulation of international movement of dogs are therefore areas that warrant further attention.

## CONFLICT OF INTEREST

The authors declare that they have no competing interests.

## AUTHOR CONTRIBUTION

**Patrick Muinde:** Conceptualization (equal); Data curation (equal); Formal analysis (equal); Investigation (equal); Project administration (equal); Writing‐review & editing (equal). **Judy Bettridge:** Conceptualization (equal); Data curation (equal); Formal analysis (equal); Investigation (equal); Methodology (equal); Supervision (equal); Visualization (equal); Writing‐review & editing (equal). **Salome Duerr:** Conceptualization (equal); Formal analysis (equal); Methodology (equal); Supervision (supporting); Visualization (equal); Writing‐review & editing (equal). **Filipe Maximiano de Sousa:** Data curation (equal); Formal analysis (equal); Methodology (equal); Visualization (equal); Writing‐review & editing (equal). **John Berezowski:** Formal analysis (equal); Methodology (equal); Writing‐review & editing (equal). **Ian Dohoo:** Formal analysis (equal); Methodology (equal); Writing‐review & editing (equal). **Titus Mutwiri:** Investigation (equal); Writing‐review & editing (equal). **Christian Odinga:** Investigation (equal); Writing‐review & editing (equal). **Eric Fevre:** Conceptualization (equal); Funding acquisition (lead); Methodology (equal); Project administration (equal); Resources (equal); Writing‐review & editing (equal). **Laura Cristina Falzon:** Conceptualization (equal); Data curation (equal); Formal analysis (equal); Investigation (equal); Methodology (equal); Project administration (equal); Supervision (equal); Writing‐original draft (equal).

## Supporting information

Supplementary Material

Supplementary Material

Supplementary Material

Supplementary Material

Supplementary Material

Supplementary Material

Supplementary Material

Supplementary Material

Supplementary Material

Supplementary Material

## Data Availability

All relevant data can be accessed through this link: https://doi.org/10.17638/datacat.liverpool.ac.uk/1230; while R code used for the analyses can be accessed through this link: https://github.com/salomeduerr/homeRange_FRDD
