# Supplementary material for: Who let the dogs out? Exploring the spatial ecology of free‐roaming domestic dogs in western Kenya
Source: Ecol Evol. 2021 Mar 20;11(9):4218–31. doi: 10.1002/ece3.7317 (PMC8093722; doi:10.1002/ece3.7317)
Supplement: Supplementary file 1 — Supplementary Material [file ECE3-11-4218-s006.docx]

**Household questions**

**Section 1: Demographic questions**

1. Date_______________ (this will Auto Insert)

2. Interviewer

1. Patrick Muinde
2. Titus Mutwiri
3. Maurice Karani
4. Kelvin Momanyi
5. Allan Ogendo
6. Joseph Ogola
7. Laura Falzon

3. Household ID [scan barcode]

4. County name

1. Busia County
2. Bungoma County
3. Kakamega County

5. Area (Location) also take GPS coordinates (coded into the form

1. Sub-county
2. Sub-location
3. Village

6. Respondent

1. Head of the household
2. Other adult
3. Minor (below 18yrs)

7. Gender of the respondent

1. Male
2. Female

8. Number of people living in the household: ________________

9. How many dogs do you own (the total number of dogs they claim responsibility of taking care of)

1. Number of males
2. Number of females

10. What are the reasons for you keeping a dog? (The assumption is that all the dogs are treated the same way; multiple options possible)

1. Hunting
2. Security (home guarding)
3. Herding (Herd dogs)
4. Pet
5. Other (Specify)

11. Do your dogs have access to the outside of the household compound?

1. Yes
2. No

12. Do your dogs have access to the human living areas?

1. Yes
2. No

13. On a typical day, for how many hours is your dog kept outside the household compound?

1. Less than 2 hours
2. 2 hours – 6 hours
3. More than 6 hours

14. Do the dogs accompany you when you go out (for work, visits, etc.)

1. Yes
2. No

15. If yes to the question above, how often? [SKIP LOGIC – this question should only be asked if the answer to Q. 14 is “Yes”)

1. Rarely (<25% of the time)
2. Sometimes ( >25% of the time)

16. Do you see other dogs apart from yours in your compound?

1. Yes
2. No

17. If yes to the question above, how often? [SKIP LOGIC – this question should only be asked if the answer to Q. 16 is “Yes”)

1. Sometimes/Occasionally
2. Frequently

18. If yes to Q. 16, how many dogs in a day? [SKIP LOGIC – this question should only be asked if the answer to Q. 16 is “Yes”)

19. Which livestock species do you keep in your household?

1. Goats
2. Sheep
3. Cattle
4. Pigs
5. None of the above

20. Can the dogs access where the livestock live? (Note: kindly observe)

1. Yes
2. No

21. How do you dispose of the dog feces?

1. Leave it
2. Dispose into the garden
3. Dispose into the toilet
4. Bury in the soil
5. Other (specify)

**Section 2: Possible transmission factors of Echinococcus**

22. Do you carry out home slaughter of your livestock?

1. Yes
2. No
3. Not applicable

23. If yes to the question above, are the carcasses inspected by a meat inspector? [SKIP LOGIC – this question should only be asked if the answer to Q. 22 is “Yes”)

1. Always
2. Sometimes
3. Never

24. How regularly do you feed your dogs (any food given to the dog)?

1. Daily
2. Every couple of days
3. Occasionally
4. Never

25. In a month, approximately how much do you spend on buying dog food (or taking care of the dog) [this should exclude left-overs, but include money for factoring dog in when preparing family meal] _________

26. Do you feed your dogs with internal organs from livestock (e.g. offals)?

1. Sometimes
2. Always
3. Never

27. If “sometimes” or “always” to the question above, where do you source the offals from? [SKIP LOGIC – this question should only be asked if the answer to Q. 26 is “Sometimes” or “Always”)

1. Own slaughter
2. Abattoir
3. From the butchery
4. Other

28. How do you prepare these offals before giving to the dogs? [SKIP LOGIC – this question should only be asked if the answer to Q. 26 is “Sometimes” or “Always”]

1. Fed raw
2. Cooked (Boiled, fried)

29. In those internal organs (offals), have you ever seen any cysts? (Show a picture) [SKIP LOGIC – this question should only be asked if the answer to Q. 26 is “Sometimes” or “Always”]

1. Yes
2. No

30. If “yes” to the question above, what do you do with the meat containing the cyst(s)? [SKIP LOGIC – this question should only be asked if the answer to Q. 29 is “Yes”]

1. Cooked and consumed in the household
2. Cooked and fed it to dogs
3. Fed to dogs raw
4. Chopped off the infected area and consume the remaining portion in the house
5. Chopped off the infected area and fed the rest as raw to dogs
6. Chopped off the infected area and fed the rest as cooked to the dogs
7. Discarded everything
8. Buried/Burned

31. What do you do with the carcasses of dead animals within your household?

1. No dead animals
2. Bury/Burn
3. Skin and eat/sell
4. Feed to dogs
5. Discard
6. Other (specify)

32. Do the dogs accompany the cattle, goats and sheep when grazing?

1. Yes
2. No
3. Don’t know

33. Do the dogs defecate where the livestock graze?

1. Yes
2. No
3. Don’t know

34. Do any of the family members play with the dogs?

1. Yes
2. No
3. Don’t know

35. Do you deworm your dogs?

1. Yes
2. No

36. If yes to the question above, how frequently do you deworm them? [SKIP LOGIC – this question should only be asked if the answer to Q. 35 is “Yes”]

1. Monthly
2. After every 3 months
3. Every 6-12 months
4. Never
5. Other (Specify)

37. If “yes” to Q. 35, which drug do you use? [SKIP LOGIC – this question should only be asked if the answer to Q. 35 is “Yes”)

38. Have you vaccinated your dogs against rabies in the last 12 months?

1. Yes
2. No

39. If yes to the question above, how much did you pay for it? [SKIP LOGIC – this question should only be asked if the answer to Q. 38 is “Yes”)

i. 0 (free through vaccination campaign)

ii. less than 100 KES

iii. 100 KES or more

iv. Do not remember

**Section 3: Proximity and access to a slaughter house/ slab**

40. Do your dogs visit the slaughter house/ slaughter slabs?

1. Yes
2. No
3. Don’t know

41. If yes to the question above, how often do they go to these slaughter houses? [SKIP LOGIC – this question should only be asked if the answer to Q. 40 is “Yes”]

1. Occasionally
2. Frequently
3. Don’t know

**Section 4: Awareness of echinococcosis amongst household members**

42. Do you know what “proglottids” are? (Show picture of proglottids)

i. Yes

ii. No

43. Have you ever seen a “proglottid” (*Echinococcus*) in dog feces?

1. Yes
2. No

44. If “yes” to the question above, what do you do when you see the “proglottids’ in the dog feces? (Multiple answers possible) [SKIP LOGIC – this question should only be asked if the answer to Q. 43 is “Yes”)

1. Nothing
2. Bury the feces
3. Dispose the feces
4. Deworm the dog
5. Other (Specify)

45. Are the offals/organs from livestock infected with cysts safe for consumption by humans and dog? (Show picture of cysts)

1. Yes
2. No
3. Don’t know

**Section 5: Sampling of the dog**

46. What is the age of this dog? (The dog to be sampled)

1. Young (less than 1 year)
2. Adult
3. Old (more than 5 years)

47. What is the sex of the dog to be sampled?

1. Male
2. Female

48. If the answer to Q. 47 is “male”, is it castrated? [SKIP LOGIC – this question should only be asked if the answer to Q. 47 is “Male”]

1. Yes
2. No

49. If the answer to Q. 47 is “female”, is it spayed/neutered? [SKIP LOGIC – this question should only be asked if the answer to Q. 47 is “Female”]

1. Yes
2. No

50. Take fecal sample and scan the barcode [SCAN BARCODE]
