# Supplementary figures and images for: Who let the dogs out? Exploring the spatial ecology of free‐roaming domestic dogs in western Kenya
Source: Ecol Evol. 2021 Mar 20;11(9):4218–31. doi: 10.1002/ece3.7317 (PMC8093722; doi:10.1002/ece3.7317)

**ZOL003818**

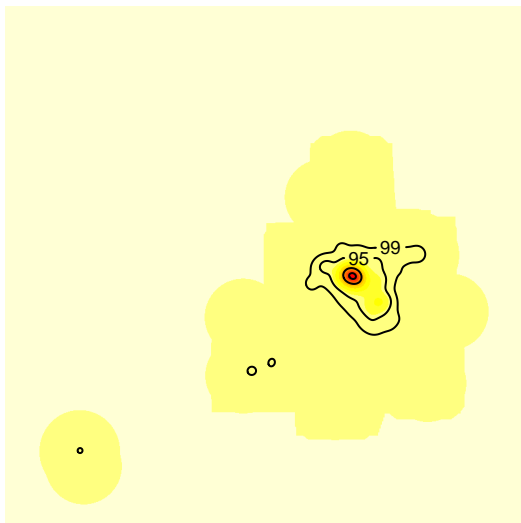

**ZOL003823**

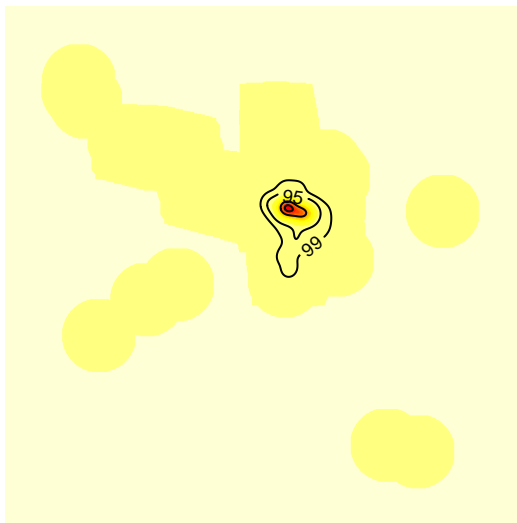

**ZOL003825**

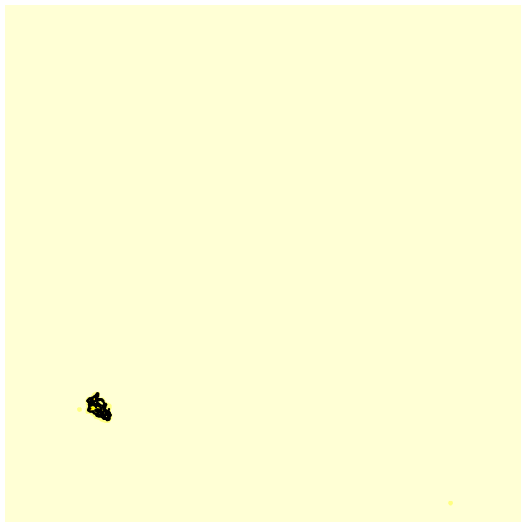

**ZOL003827**

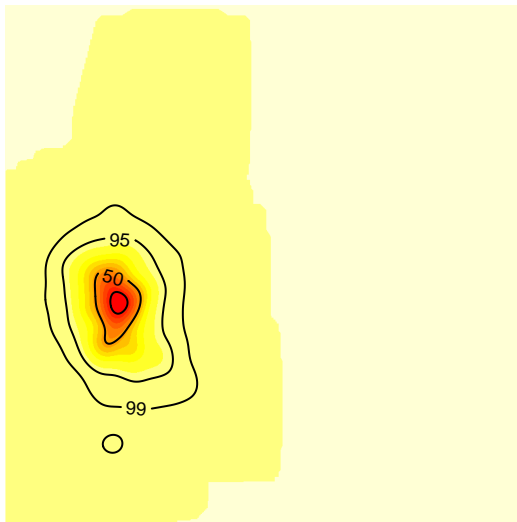

**ZOL003829**

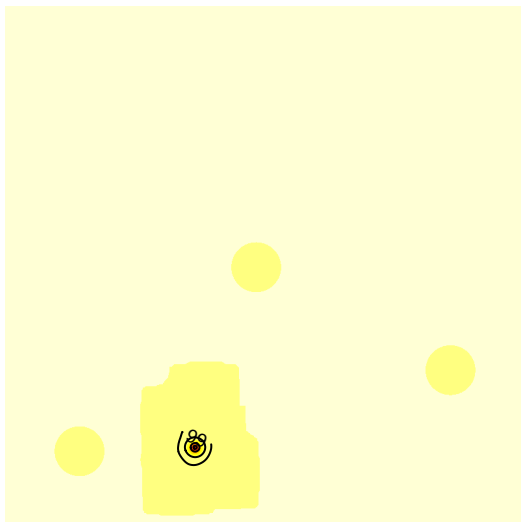

**ZOL003831**

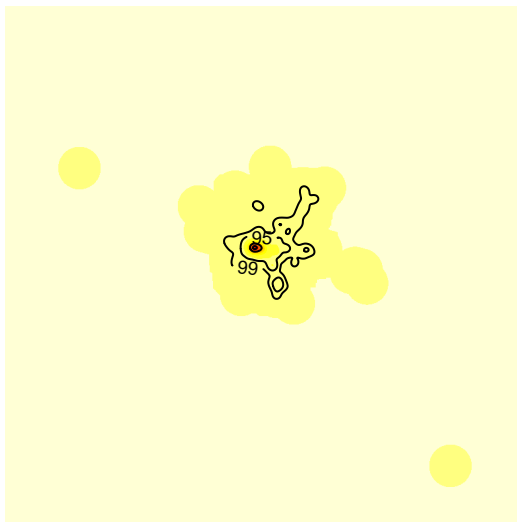

**ZOL003833**

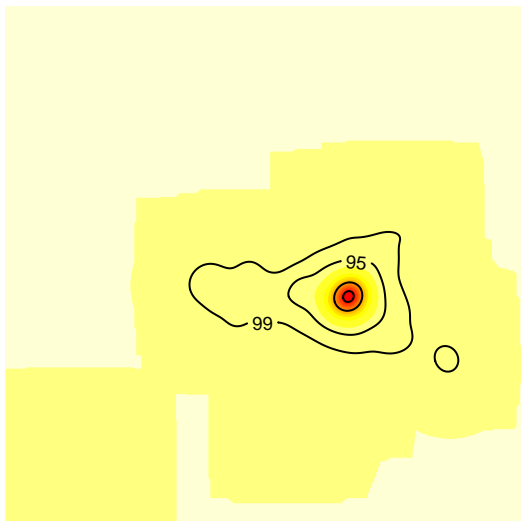

**ZOL004186**

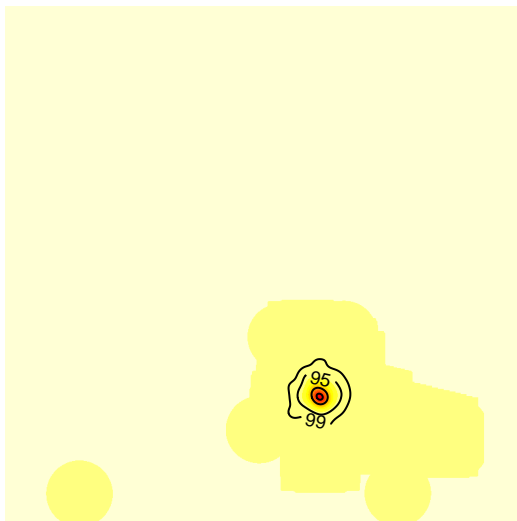

# volumes of BRB UD<sub>s</sub>, Amerikwai

ZOL004188

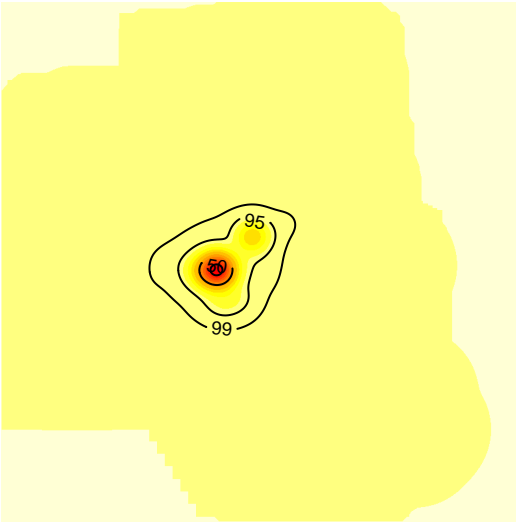

Supplement: Supplementary file 2 — Supplementary Material [file ECE3-11-4218-s007.pdf]

**ZOL003766**

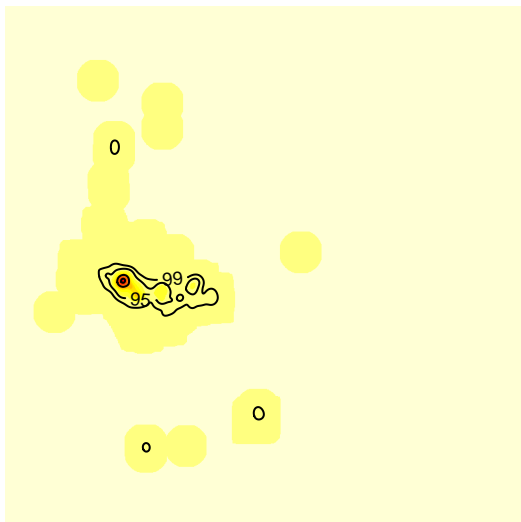

**ZOL003764**

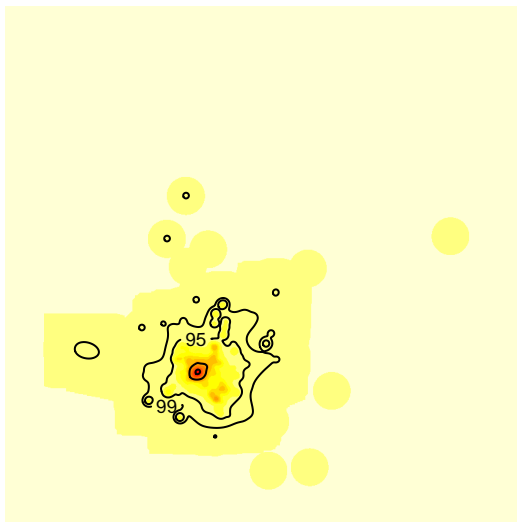

**ZOL003762**

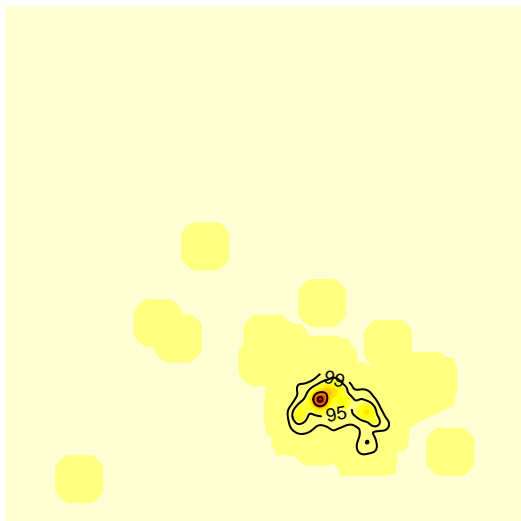

**ZOL003773**

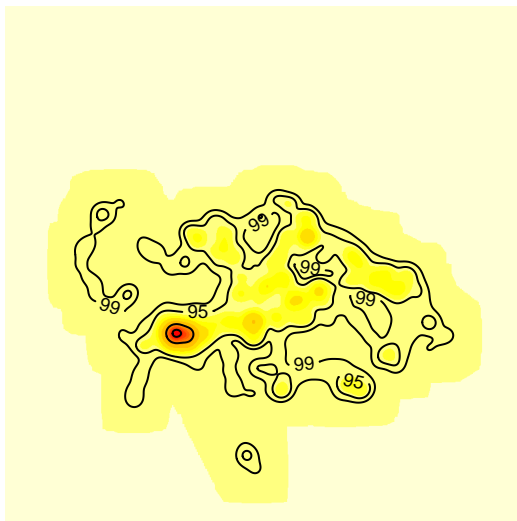

**ZOL003769**

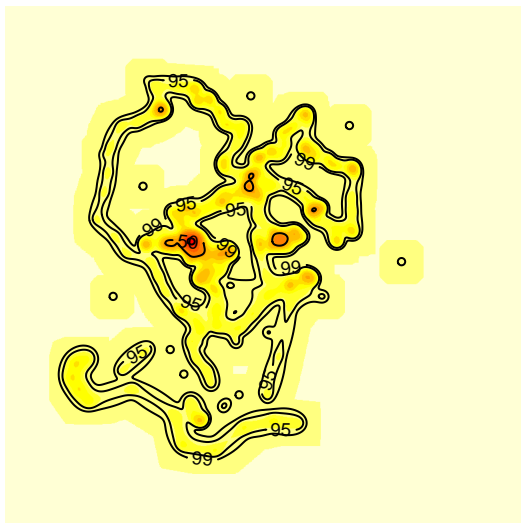

**ZOL003767**

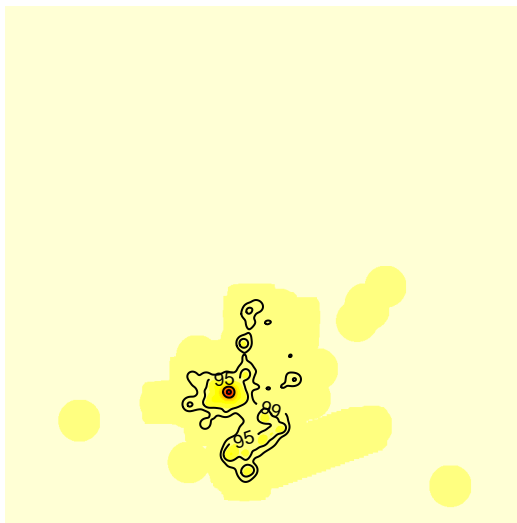

**ZOL003775**

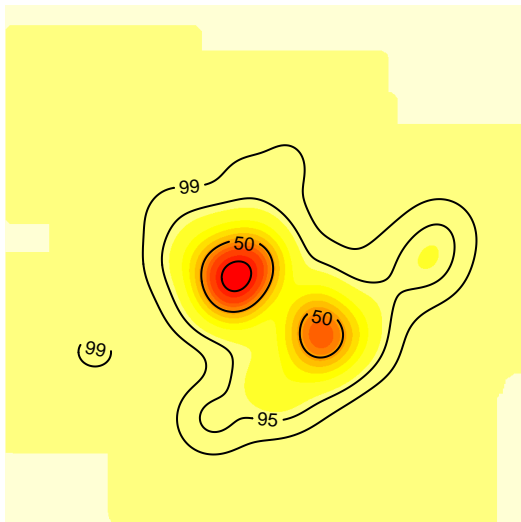

**ZOL003777**

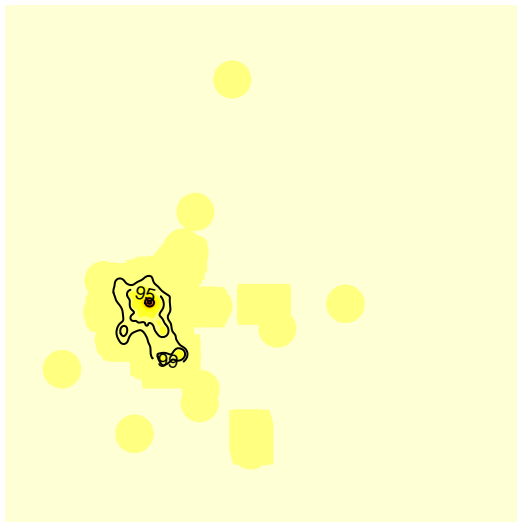

# volumes of BRB UDs,Amukura

ZOL003779

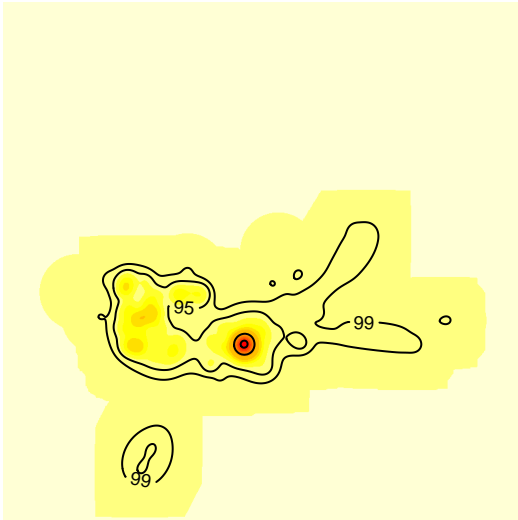

Supplement: Supplementary file 3 — Supplementary Material [file ECE3-11-4218-s003.pdf]

**ZOL004270**

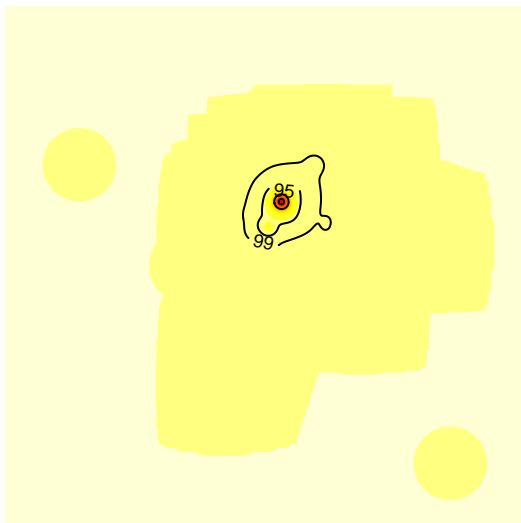

**ZOL004274**

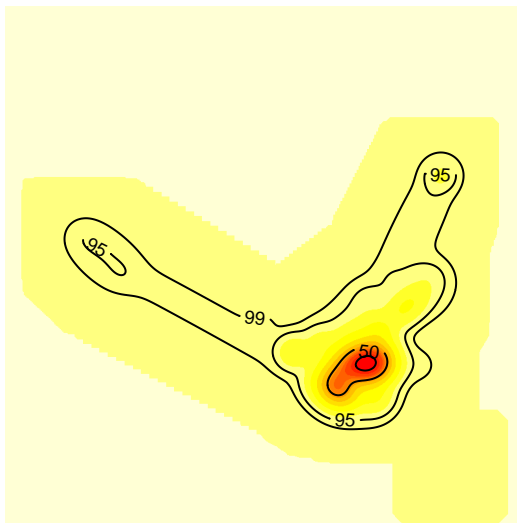

**ZOL004314**

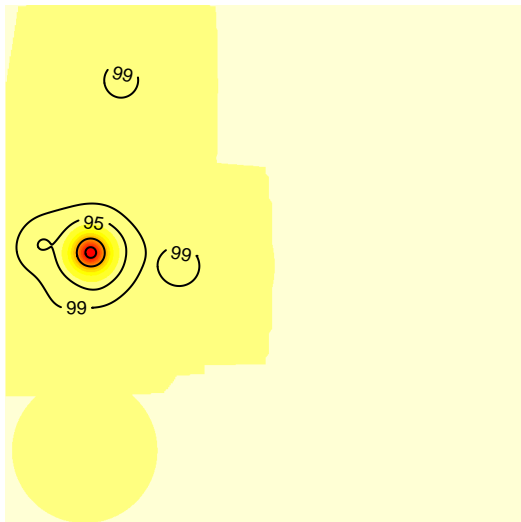

**ZOL004275**

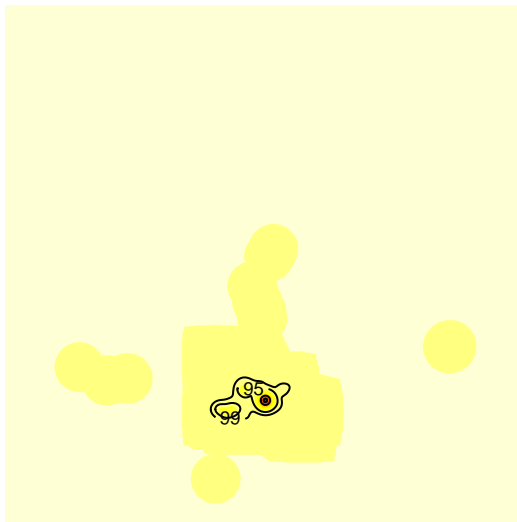

**ZOL004277**

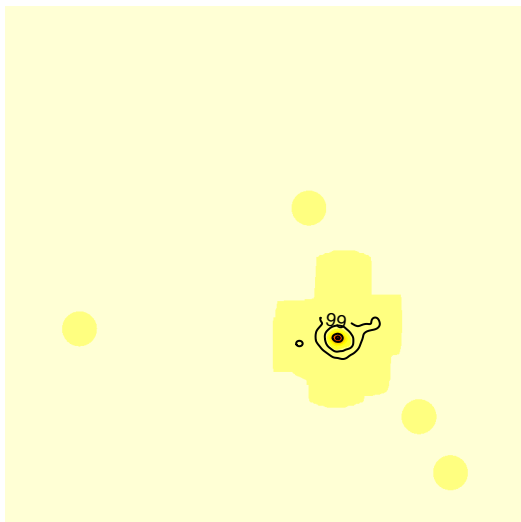

**ZOL004279**

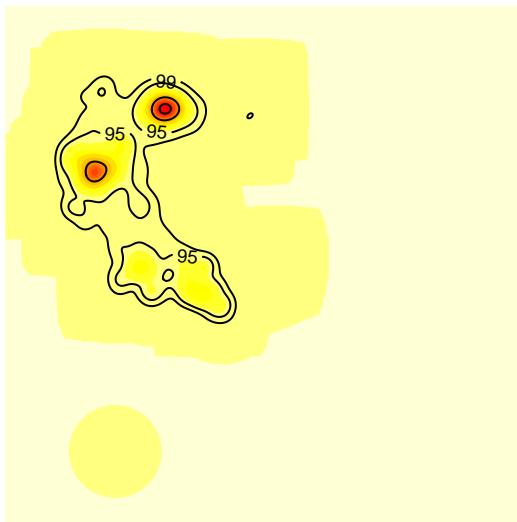

**ZOL004281**

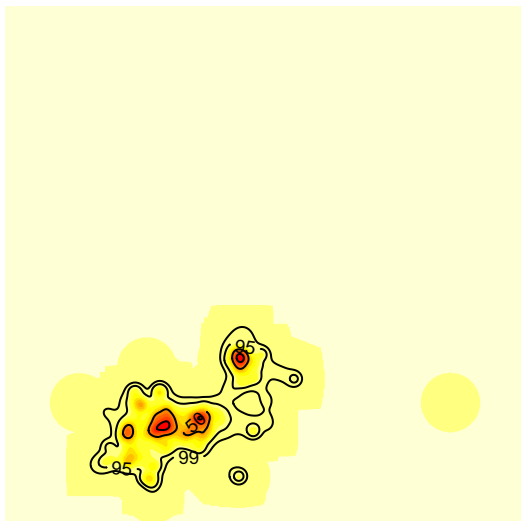

**ZOL004316**

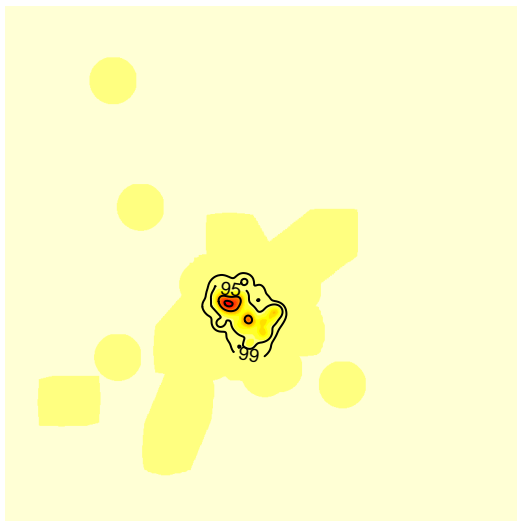

# volumes of BRB UDs,Bumala

ZOL004318

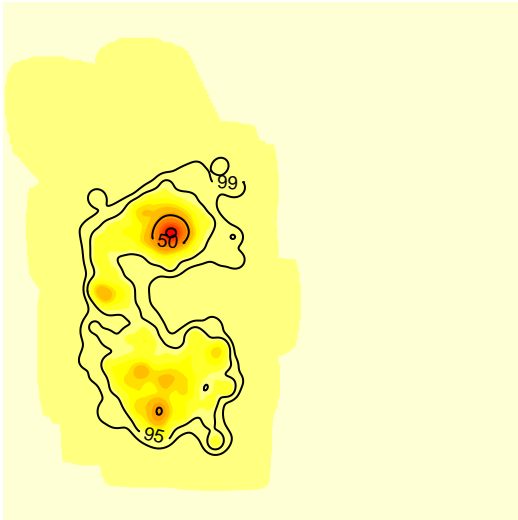

ZOL004283

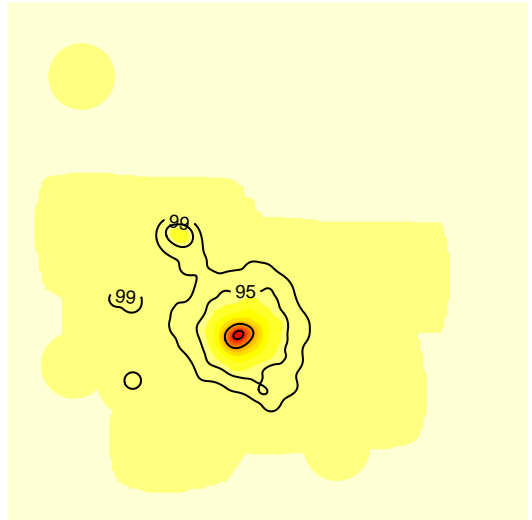

Supplement: Supplementary file 4 — Supplementary Material [file ECE3-11-4218-s009.pdf]

**ZOL003837**

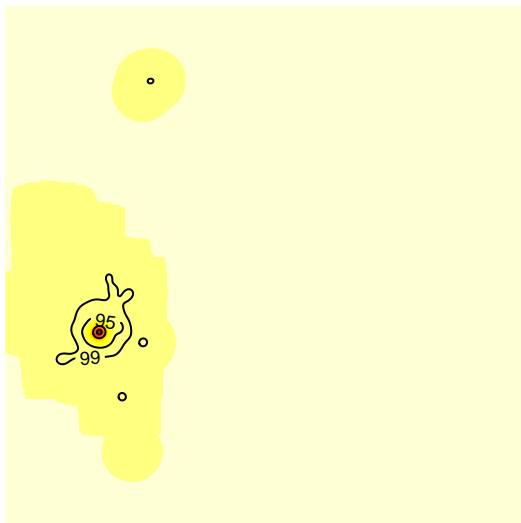

**ZOL003839**

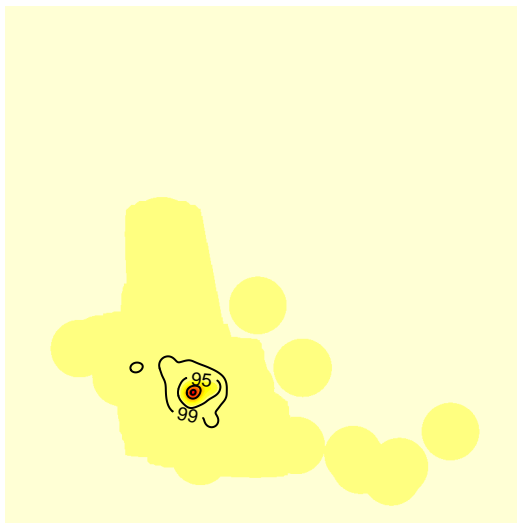

**ZOL003841**

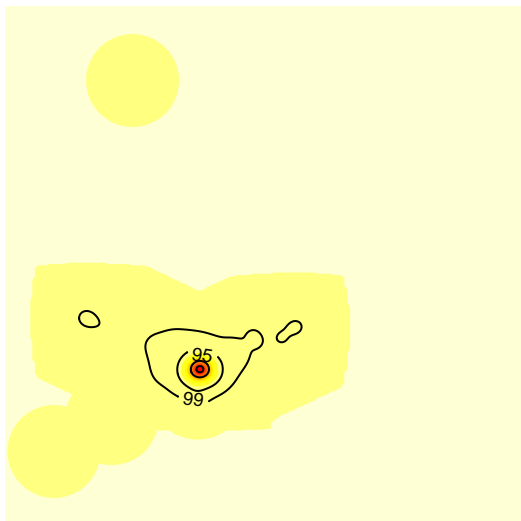

**ZOL003846**

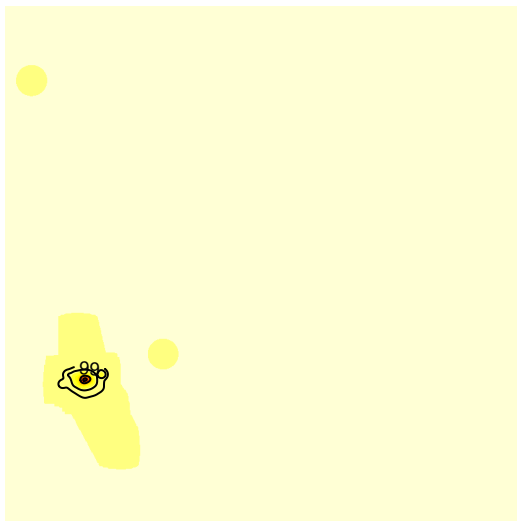

**ZOL003848**

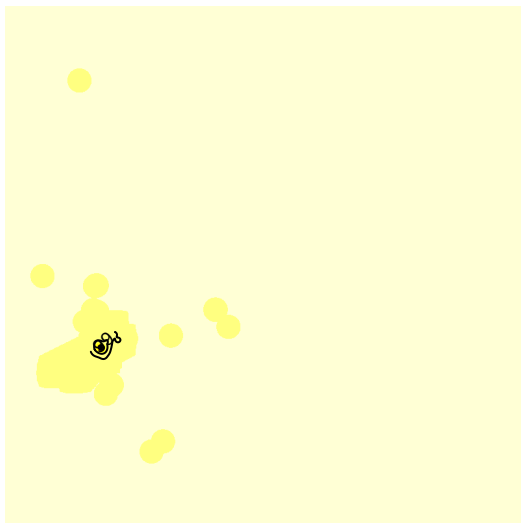

**ZOL003852**

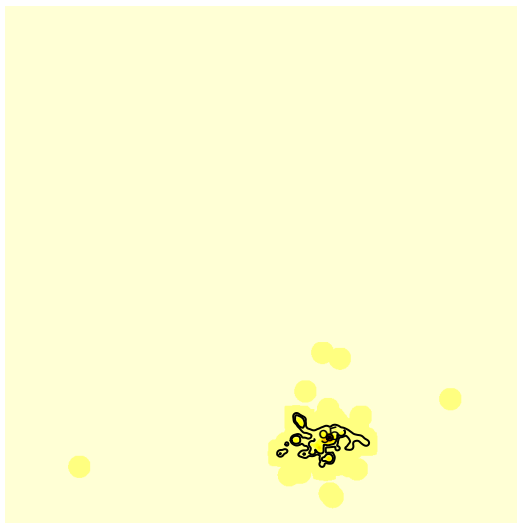

**ZOL003854**

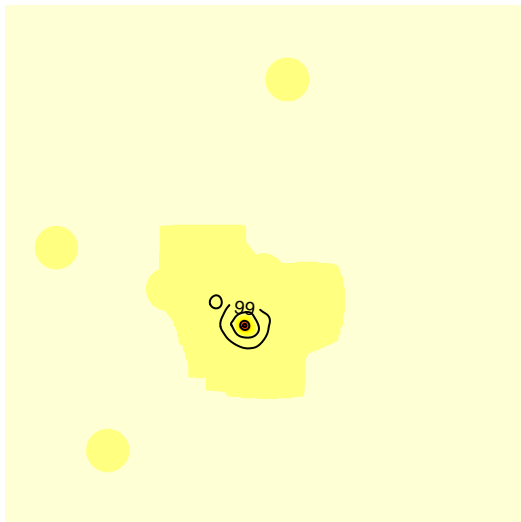

**ZOL003859**

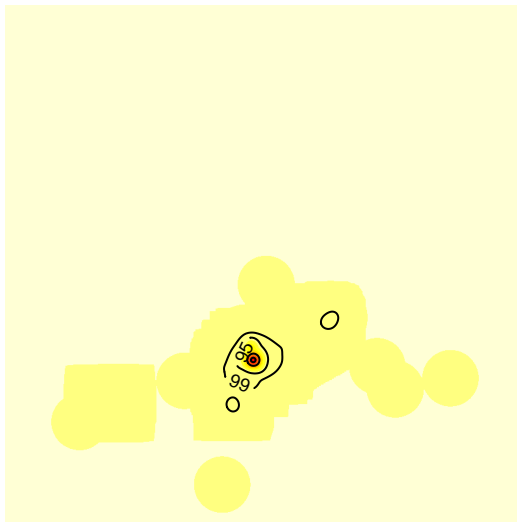

# volumes of BRB UD<sub>s</sub>, Busia

ZOL004183

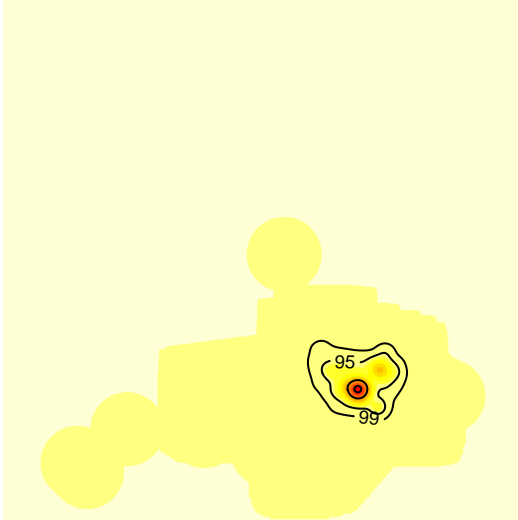

ZOL004174

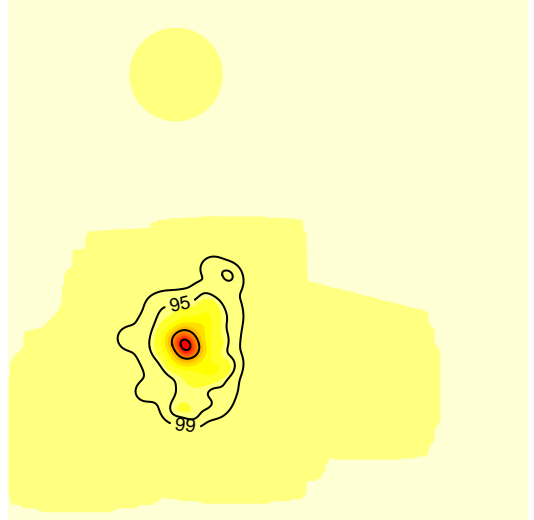

Supplement: Supplementary file 5 — Supplementary Material [file ECE3-11-4218-s002.pdf]

**ZOL003781**

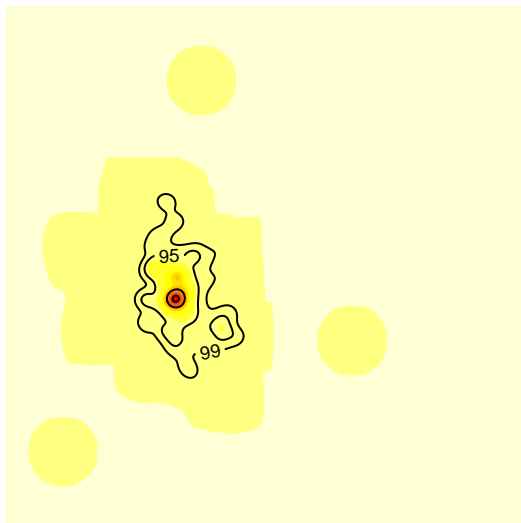

**ZOL003783**

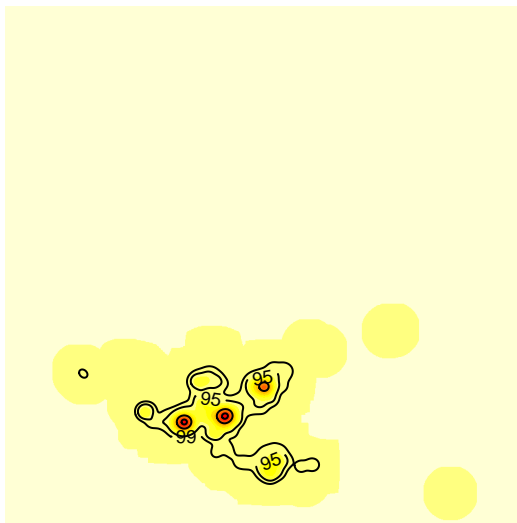

**ZOL003785**

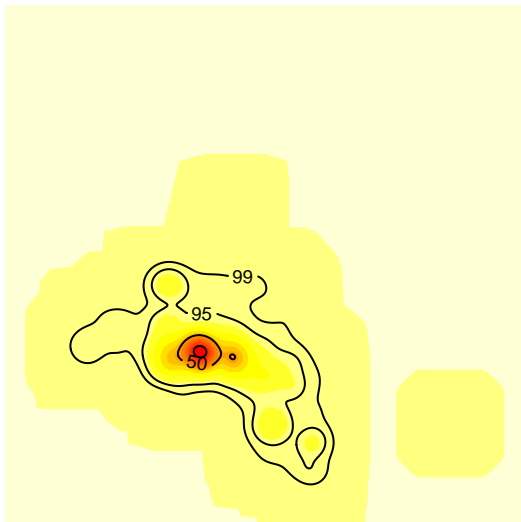

**ZOL003787**

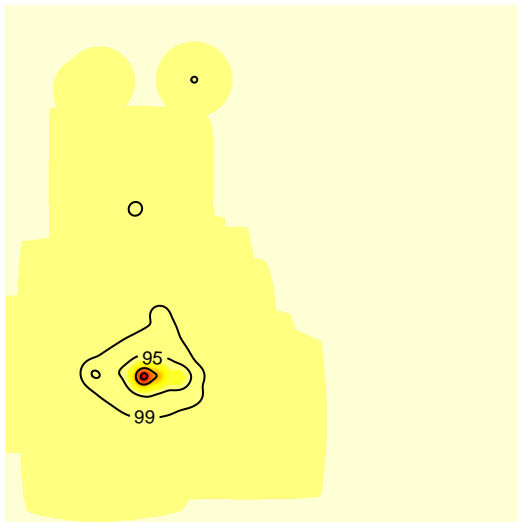

**ZOL003789**

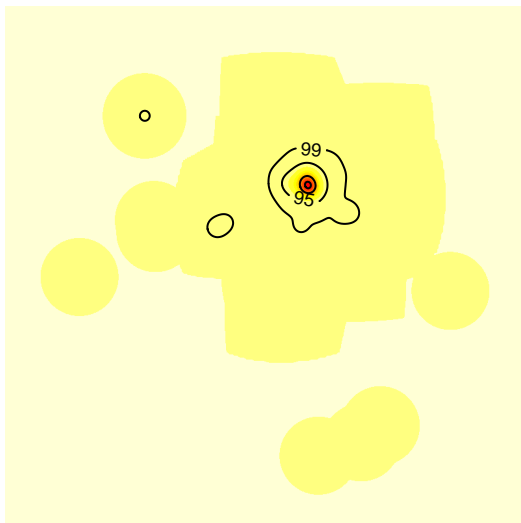

**ZOL003791**

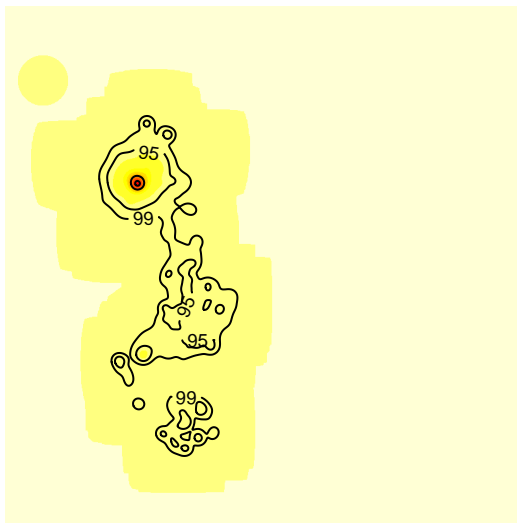

**ZOL003793**

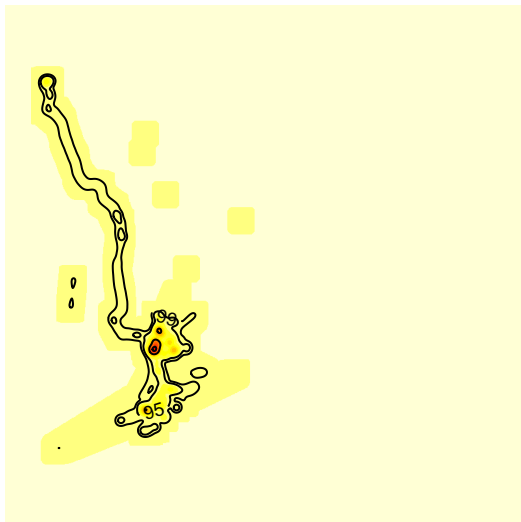

**ZOL003795**

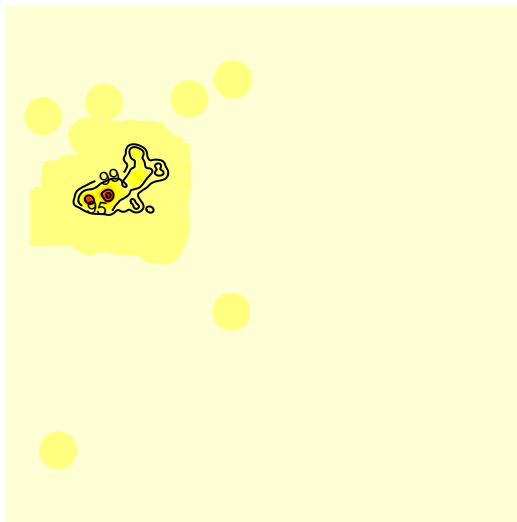

# volumes of BRB UD<sub>s</sub>, Funyula

ZOL003797

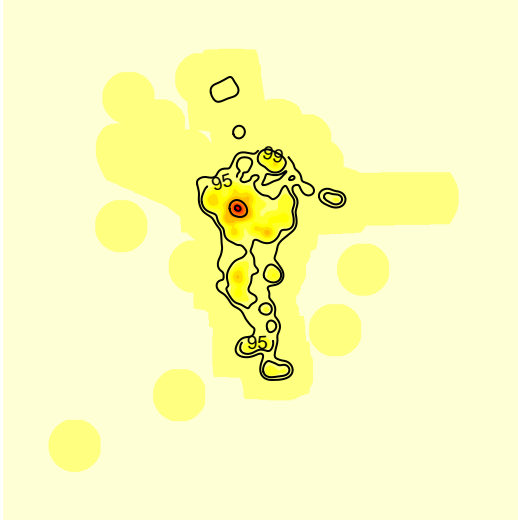

ZOL003799

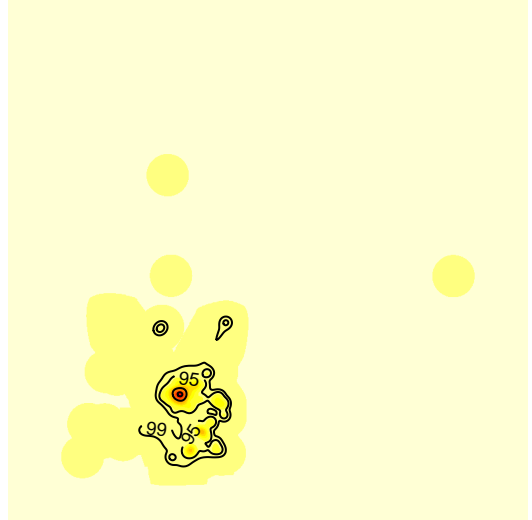

Supplement: Supplementary file 6 — Supplementary Material [file ECE3-11-4218-s001.pdf]

**ZOL002766**

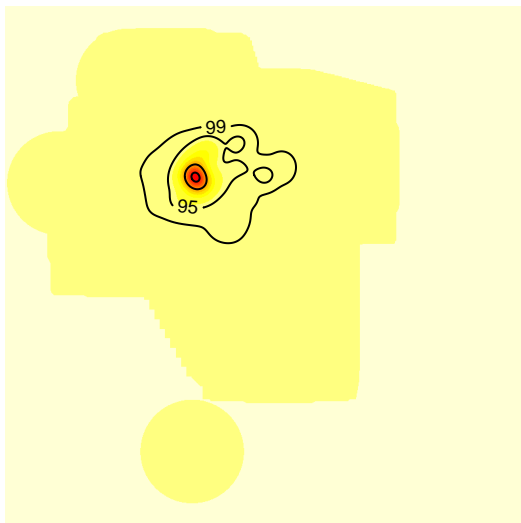

**ZOL002770**

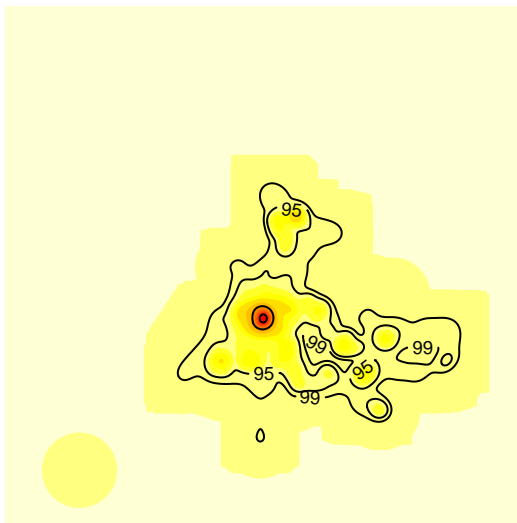

**ZOL002768**

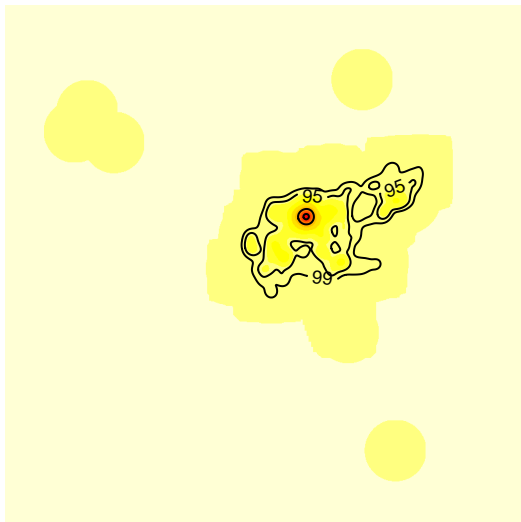

**ZOL002772**

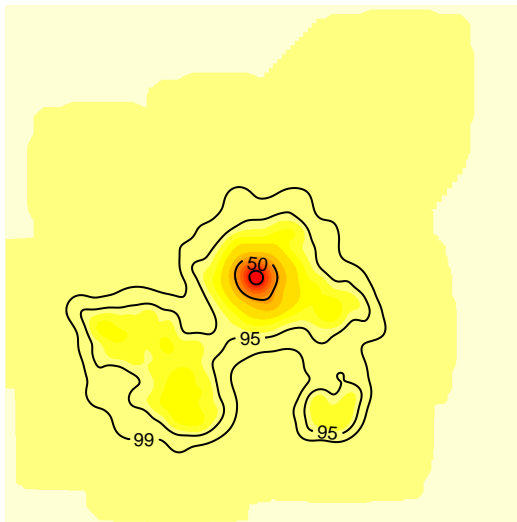

# volumes of BRB UD<sub>s</sub>,Malaba

ZOL002774

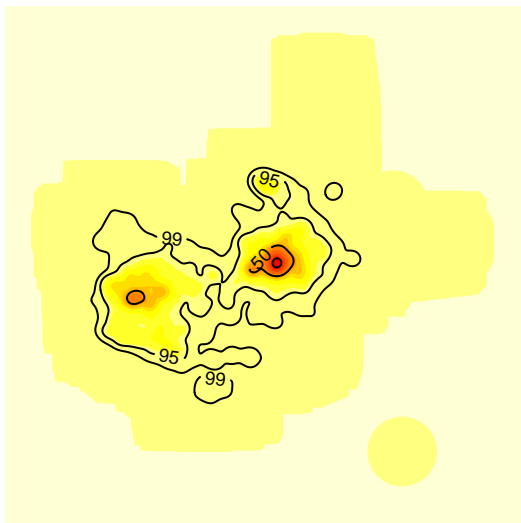

ZOL002776

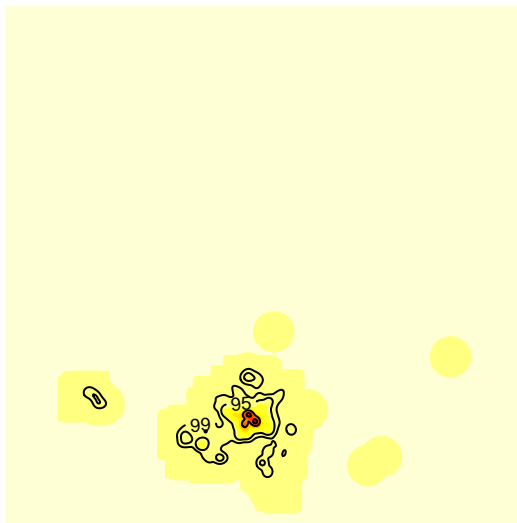

ZOL002780

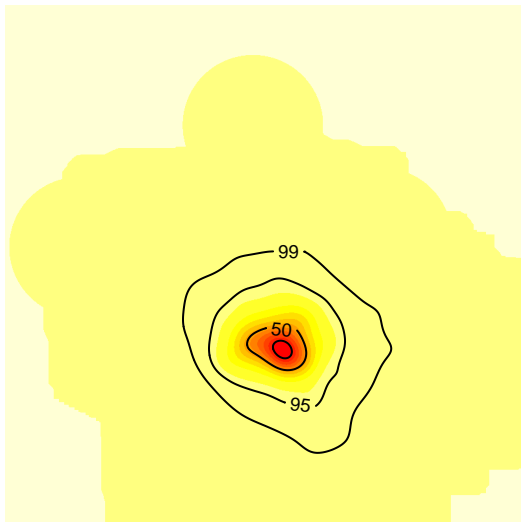

ZOL002782

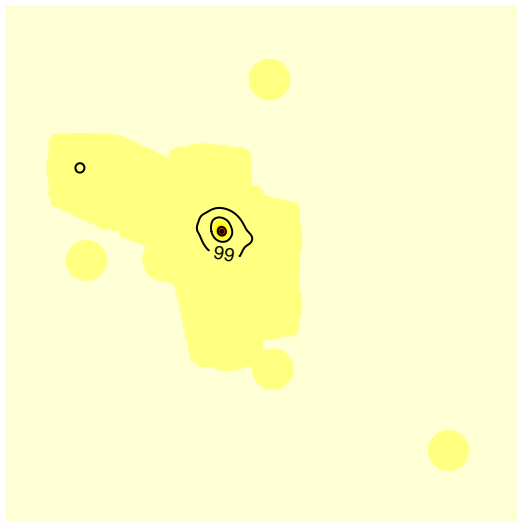

Supplement: Supplementary file 7 — Supplementary Material [file ECE3-11-4218-s010.pdf]

**ZOL002745**

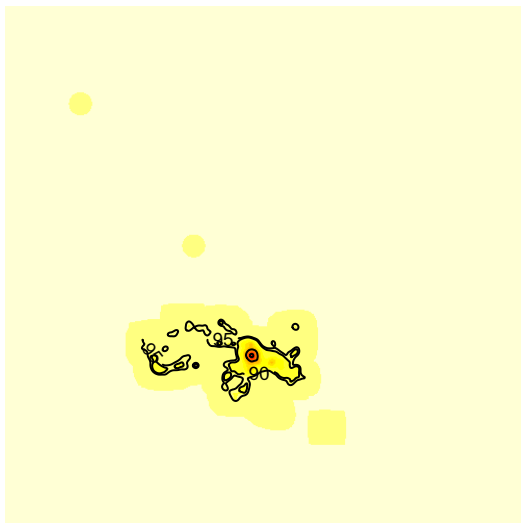

**ZOL002747**

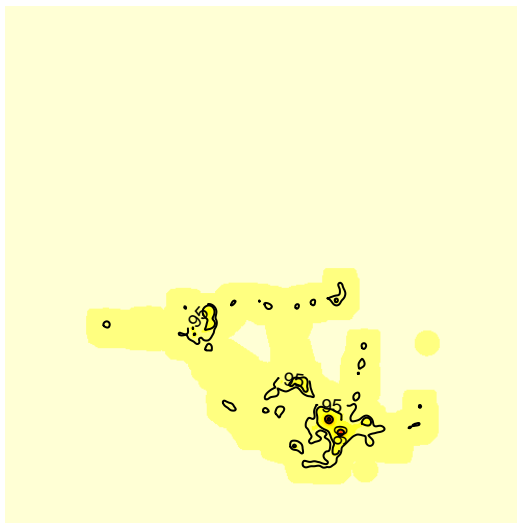

**ZOL002749**

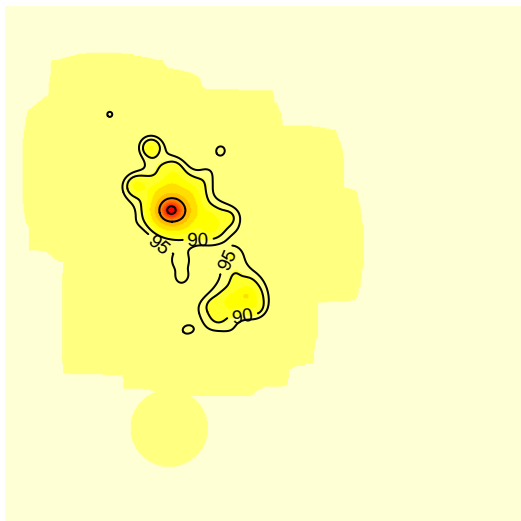

**ZOL002751**

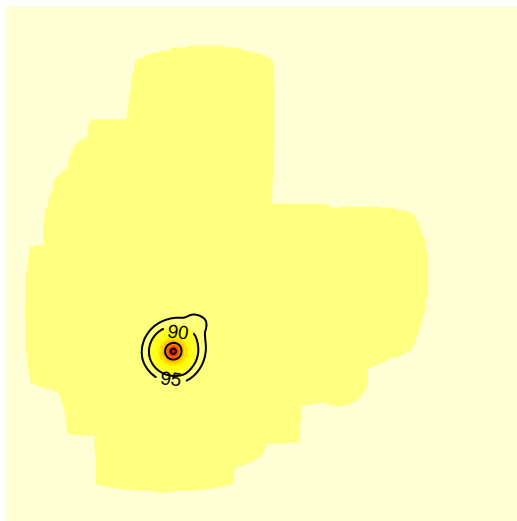

**ZOL002753**

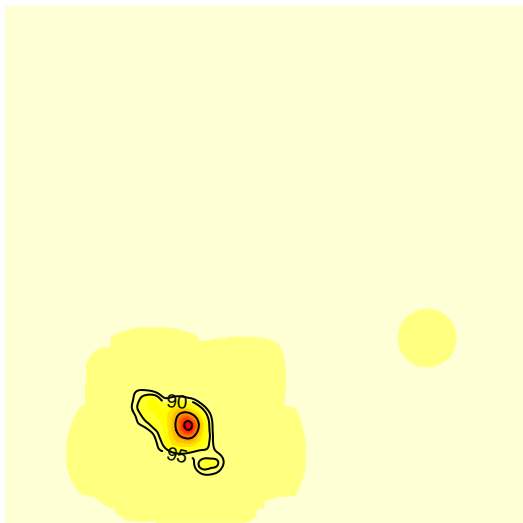

**ZOL002758**

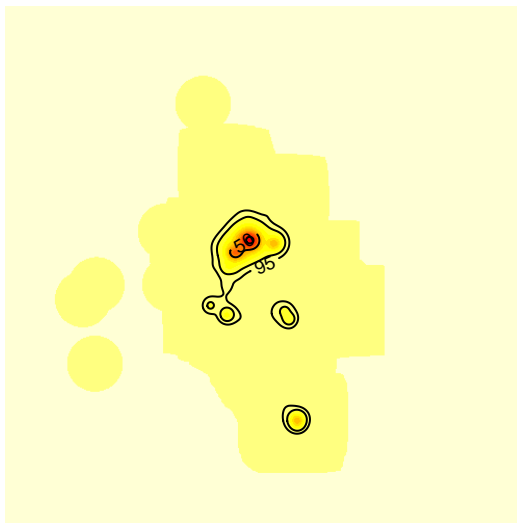

**ZOL002760**

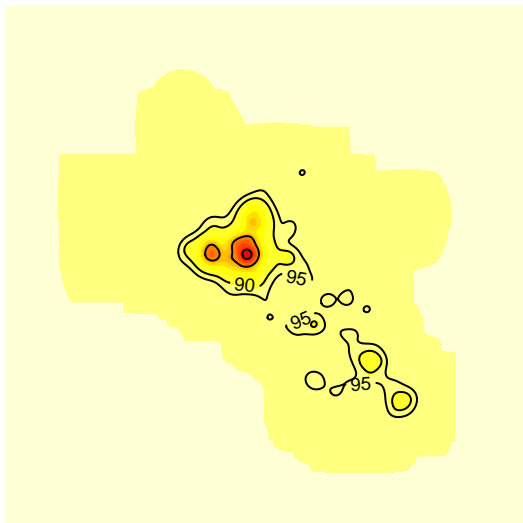

**ZOL002762**

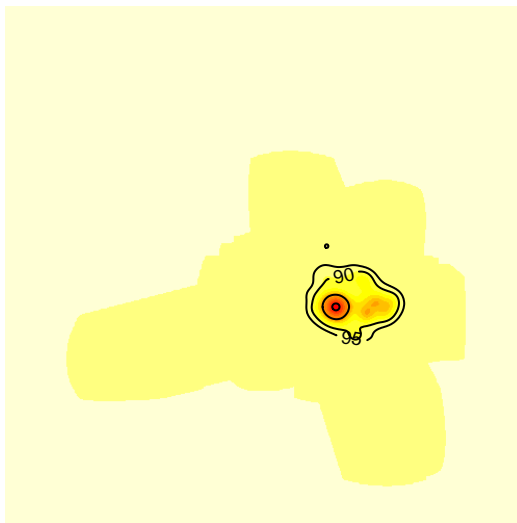

# volumes of BRB UD's, Mudembi

ZOL002764

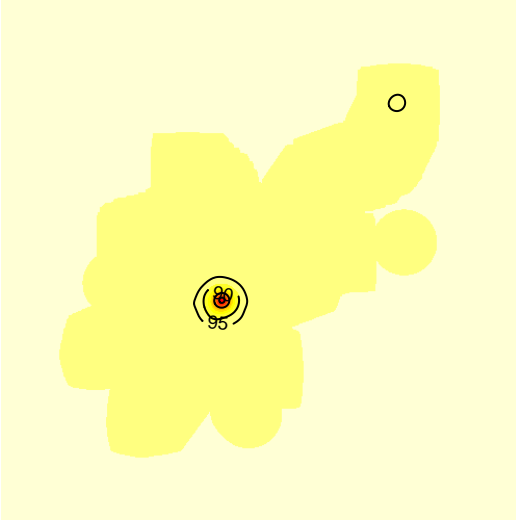

Supplement: Supplementary file 8 — Supplementary Material [file ECE3-11-4218-s004.pdf]

**ZOL003753**

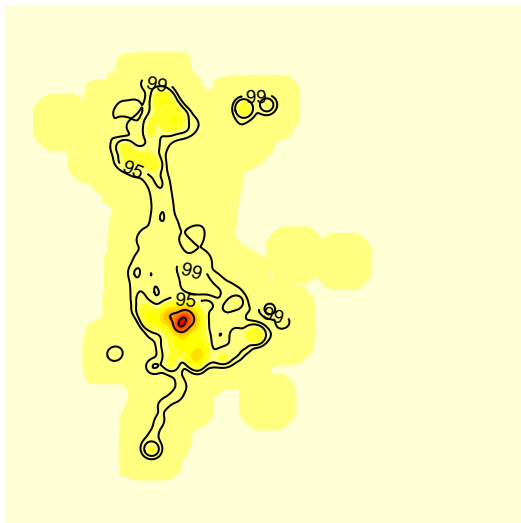

**ZOL003756**

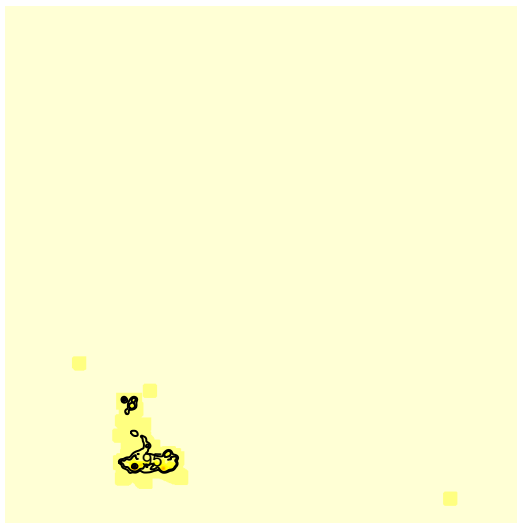

**ZOL002795**

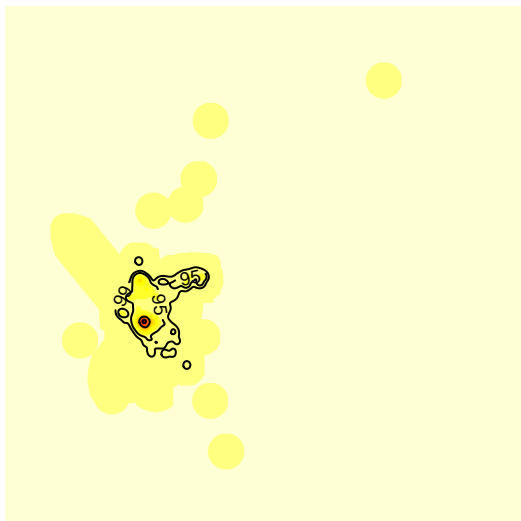

**ZOL003758**

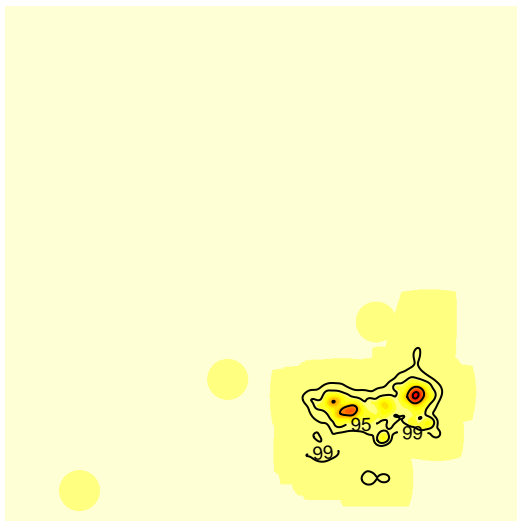

**ZOL003760**

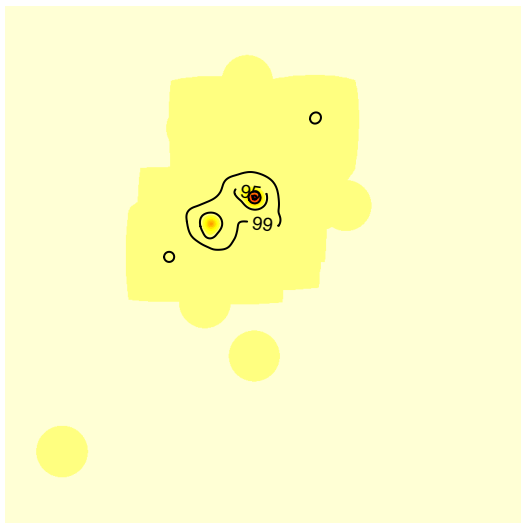

**ZOL002788**

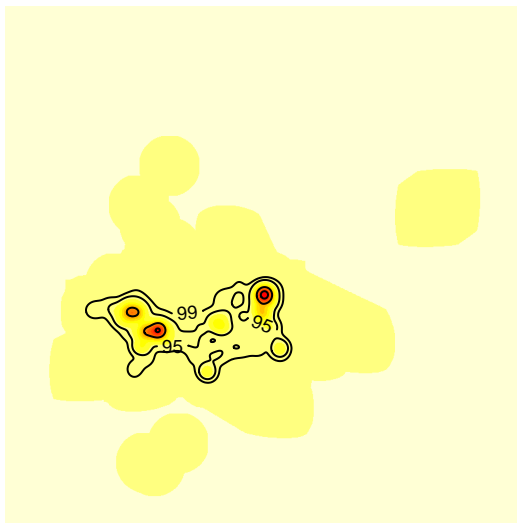

**ZOL002786**

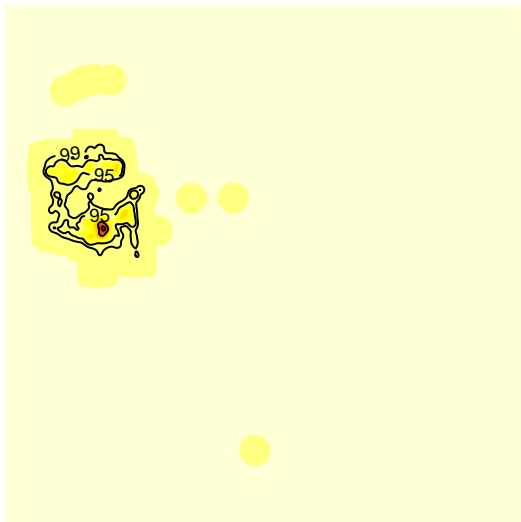

**ZOL002790**

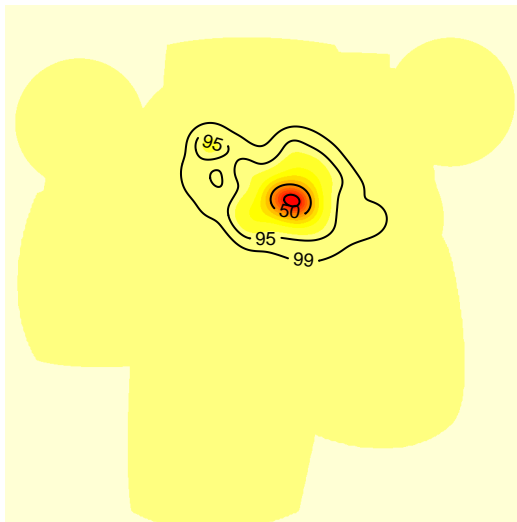

# volumes of BRB UD's,Nambale

ZOL004288

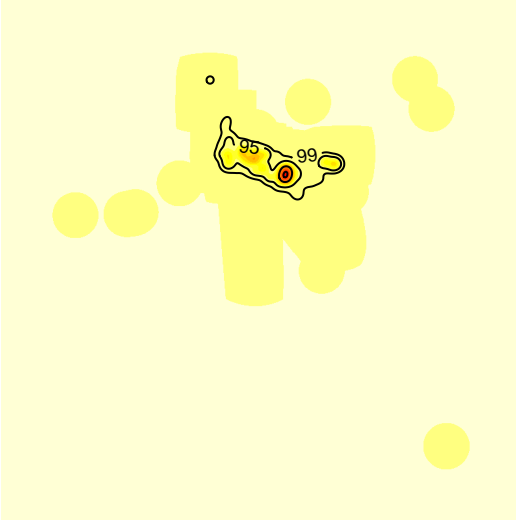

Supplement: Supplementary file 9 — Supplementary Material [file ECE3-11-4218-s008.pdf]
