# Supplementary material for: Who let the dogs out? Exploring the spatial ecology of free‐roaming domestic dogs in western Kenya
Source: Ecol Evol. 2021 Mar 20;11(9):4218–31. doi: 10.1002/ece3.7317 (PMC8093722; doi:10.1002/ece3.7317)
Supplement: Supplementary file 10 — Supplementary Material [file ECE3-11-4218-s005.docx]

**S1.** The locations corresponding to centroid points for the 10%, 50% and 90% isopleths obtained with the utilization distribution method for 73 dogs tagged in 8 sites in Busia County.

1. Amerikwai

|  | **10%** |  | **50%** |  | **90%** |  |
| --- | --- | --- | --- | --- | --- | --- |
| **ID** | **Isopleth area** | **Site at centroid** | **Isopleth area** | **Site at centroid** | **Isopleth area** | **Site at centroid** |
| 3818 | 696.05 | Dog’s household | 6242.01 | Dog's household | 43998.57 | Dog’s household |
| 3823 | 914.12 | Dog's household | 7799.79 | Dog's household | 54240.49 | Dog's household |
| 3825 | 813.35 | Dog's household | 652.71 | Human compound | 10177.36 | A human compound |
|  |  |  | 10659.34 | Dog's household | 170739.0 | Small rubbish dump next to a human compound |
|  |  |  |  |  | 1498.93 | Rubbish dump next to a human compound |
|  |  |  |  |  | 35499.95 | Grassy area in front of a human compound |
|  |  |  |  |  | 853.31 | Rubbish dump between human compounds |
|  |  |  |  |  | 19917.16 | Grassy area within a human compound |
|  |  |  |  |  | 10393.57 | A dump site within a human compound (owner confirmed that she has a male dog and other dogs often come to her house) |
|  |  |  |  |  | 5586.22 | Grassy area by the side of the main road, in front of an egg depot and butchery |
|  |  |  |  |  | 3706.54 | Human compound right next to the slaughter slab |
| 3827 | 1837.46 | Dog's household | 14291.38 | Dog's household | 55495.37 | Grassy area within the dog's household |
| 3829 | 537.12 | Manure pile within the dog's household | 5064.03 | Dog's household | 40928.43 | Dog's household |
| 3831 | 774.82 | Dog's household | 7431.42 | Dog's household | 57500.78 | Dog's household |
| 3833 | 565.77 | Dog's household | 4356.88 | Dog's household | 25099.74 | Dog's household |
| 4186 | 813.64 | Dog’s household | 6398.20 | Dog's household | 30845.69 | Dog's household |
| 4188 | 705.40 | Dog's household | 6431.71 | Dog's household | 34289.70 | Dog's household |

1. Amukura

|  | **10%** |  | **50%** |  | **90%** |  |
| --- | --- | --- | --- | --- | --- | --- |
| **ID** | **Isopleth area** | **Site at centroid** | **Isopleth area** | **Site at centroid** | **Isopleth area** | **Site at centroid** |
| 3766 | 748.89 | Dog’s household | 7858.99 | Dog’s household | 63350.72 | Human compound down the road from dog’s household (house owner confirmed that the neighbour’s dog comes to their compound) |
|  |  |  |  |  | 2730.41 | Small rubbish dump next to food market |
| 3764 | 2171.06 | Dog's household | 37849.84 | Dog's household | 251867.90 | Within a human compound (owner confirms that she has dogs) |
|  |  |  |  |  | 375.18 | Field next to a human compound |
|  |  |  |  |  | 907.72 | Close to the remains of a mud house inside a human compound |
| 3762 | 841.24 | Dog's household | 8476.99 | Dog's household | 62619.16 | Field just outside the dog's household |
| 3773 | 909.46 | Dog's household (owner confirmed that the dog is still alive and likes to roam in fields) | 8145.97 | Dog's household | 139295.50 | Maize field |
|  |  |  |  |  | 4044.25 | Open space close to an eating point and children’s play area |
|  |  |  |  |  | 9395.95 | Field by the road |
|  |  |  |  |  | 346.91 | Maize field |
|  |  |  |  |  | 993.04 | Human compound (owners said they do not have any dogs) |
|  |  |  |  |  | 6347.98 | Area between human rental houses |
|  |  |  |  |  | 28310.43 | Maize field |
| 3769 | 1321.13 | Dog's household | 23206.75 | Dog's household | 33047.27 | Human compound far from the dog's household |
|  |  |  | 2482.11 | Path between maize and cassava field | 48377.96 | Human compound (they said they have a dog and other dogs come here with goat herds) |
|  |  |  | 7680.60 | Maize field | 25.63 | Maize field |
|  |  |  |  |  | 4526.54 | Maize field |
|  |  |  |  |  | 225.70 | Bush by a dirt road |
|  |  |  |  |  | 385.80 | Human compound |
|  |  |  |  |  | 1506.79 | Garden beside human compound |
|  |  |  |  |  | 2031.85 | Sweet potato field |
|  |  |  |  |  | 485979.10 | In front of a human compound |
|  |  |  |  |  | 48627.67 | Sugarcane field |
|  |  |  |  |  | 9544.15 | Bush along a small path (observed dogs defecating in this area) |
|  |  |  |  |  | 11325.68 | Forest |
|  |  |  |  |  | 38941.52 | Field next to a church |
|  |  |  |  |  | 4143.89 | Maize field by a footpath |
|  |  |  |  |  | 87063.23 | Field close to a dirt road |
|  |  |  |  |  | 28210.52 | Sugarcane field |
|  |  |  |  |  | 21982.22 | Bush by the road |
|  |  |  |  |  | 40444.13 | Open field next to road |
|  |  |  |  |  | 28483.73 | Maize field |
| 3767 | 830.14 | Dog's household | 7476.97 | Dog's household | 61210.72 | Dog’s household |
|  |  |  |  |  | 12442.74 | Human compound |
|  |  |  |  |  | 3610.34 | In front of the slaughterhouse |
|  |  |  |  |  | 223.53 | Garden in a human compound (owners said they do not have dogs, but they see dogs come to eat avocadoes that drop from their avocado tree) |
|  |  |  |  |  | 3684.09 | Fish and omena market |
|  |  |  |  |  | 1623.31 | Jiko spot where they cook in a rental compound |
|  |  |  |  |  | 745.57 | Rubbish dump close to livestock market |
| 3775 | 911.51 | Dog's household | 5411.27 | Dog's household | 29762.09 | Grassy area within dog's household (observed dog lying here) |
|  |  |  | 2132.12 | Human compound close to dog's household |  |  |
| 3777 | 624.44 | Dog's household | 6203.11 | Dog's household | 58809.49 | Dog's household |
|  |  |  |  |  | 1086.82 | Human compound |
|  |  |  |  |  | 9295.27 | Field next to a human compound |
| 3779 | 903.15 | Dog's household | 10152.19 | Dog's household | 144800.60 | Field in front of a human compound (owner confirmed that she has a female dog and often sees other dogs in her household) |
|  |  |  |  |  | 3710.68 | Field next to a human compound |
|  |  |  |  |  | 2493.20 | Sugarcane field |

1. Bumala

|  | **10%** |  | **50%** |  | **90%** |  |
| --- | --- | --- | --- | --- | --- | --- |
| **ID** | **Isopleth area** | **Site at centroid** | **Isopleth area** | **Site at centroid** | **Isopleth area** | **Site at centroid** |
| 4270 | 550.78 | Rubbish dump | 5082.67 | Rubbish dump | 42575.34 | Chicken coop next to rubbish dump |
| 4274 | 1115.51 | Kennel in dog’s household | 8446.73 | Dog's household | 43757.94 | In front of a mandazi stall right next to the dog's household |
| 4314 | 723.11 | Dog's household | 6099.18 | Dog's household | 38030.36 | Dog's household |
| 4275 | 360.99 | Dog's household | 3174.12 | Dog's household | 5665.15 | Within a human compound |
|  |  |  |  |  | 27530.67 | Dog's household |
| 4277 | 872.27 | Area under tree in front of  dog's household | 7906.40 | Area under tree in front of dog's household | 39798.69 | Area under a tree in front of the dog's household |
| 4279 | 1053.01 | Open area in front of dog's household | 4392.16 | Uninhabited store rooms within a rental compound across the road (dog owners confirmed that dog likes to visit this area) | 34662.58 | Uninhabited store rooms within a rental compound across the road |
|  |  |  | 6506.53 | Area in front of the dog's household | 6686.65 | Big rubbish dump across the road |
|  |  |  |  |  | 28208.88 | Maize field |
|  |  |  |  |  | 13121.41 | In the middle of a crossroad |
| 4281 | 3683.97 | Dog's household (owner confirmed that the dog likes to roam) | 1275.15 | Rubbish dump below road culvert | 2055.05 | Ditch next to the road |
|  | 171.50 | Human compound far from dog's household (across valley and river) | 357.07 | Within a human compound under an avocado tree | 130338.90 | Just outside the dog's household |
|  |  |  | 29745.99 | Pit latrine in a small maize field | 32897.20 | Field far from the dog's household |
|  |  |  | 7070.21 | Human compound far from dog's household |  |  |
| 4316 | 1442.93 | Dog's household | 11900.98 | Dog's household | 72101.87 | Human compound |
|  |  |  | 1823.53 | Maize field |  |  |
| 4318 | 1362.65 | Dog's household | 18513.92 | Dog's household | 99635.80 | Maize field in front of a human compound |
|  |  |  |  |  | 100821.80 | In front of a retail shop by the side of the main road (owners confirmed that the dog likes to visit this area) |
|  |  |  |  |  | 667.83 | By the side of the main road where matatus park here (observed passengers throwing rubbish out of the windows; owners confirmed that their dog likes to visit this area) |
| 4283 | 1730.06 | Dog's household | 14667.04 | Dog's household | 1440.28 | Grassy area next to a church further down the road (people present confirmed that food services are regularly held in this spot) |
|  |  |  |  |  | 88555.28 | Dog's household |

1. Busia

|  | **10%** |  | **50%** |  | **90%** |  |
| --- | --- | --- | --- | --- | --- | --- |
| **ID** | **Isopleth area** | **Site at centroid** | **Isopleth area** | **Site at centroid** | **Isopleth area** | **Site at centroid** |
| 3837 | 543.80 | Dog's household | 4854.03 | Dog's household | \| 35715.31 \| \| --- \| \|  \| | Dog's household |
| 3839 | 853.70 | Dog's household | 8390.72 | Dog's household | 58596.35 | Outside dog's household |
| 3841 | 626.57 | Dog's household | 5043.01 | Dog's household | 31509.42 | Dog's household |
| 3846 | 963.81 | Dog's household | 9185.91 | Dog's household | 48115.91 | Dog's household |
| 3848 | 549.14 | Dog's household | 7662.44 | Dog's household | 62185.06 | Dog's household |
| 3852 | 527.09 | Dog's household | 7394.32 | Dog's household | 5063.874 | Open area where livestock is grazed |
|  |  |  |  |  | 7067.99 | Small maize field next to human rental compounds |
|  |  |  |  |  | 61435.79 | Grassy area next to a human compound |
|  |  |  |  |  | 7040.12 | Human compound quite far from the dog's household |
| 3854 | 583.96 | Human rental compounds | 5480.44 | Human rental compounds | 33275.05 | Human rental compounds |
| 3859 | 667.97 | Dog's household | 5801.89 | Dog's household | 30999.08 | Dog's household |
| 4183 | 811.75 | Dog's household | 8878.42 | Dog's household | 54938.97 | Human compound right next to dog's household |
| 4174 | 1273.65 | Dog's household | 11109.66 | Dog's household | 62137.26 | Dog's household |

1. Funyula

|  | **10%** |  | **50%** |  | **90%** |  |
| --- | --- | --- | --- | --- | --- | --- |
| **ID** | **Isopleth area** | **Site at centroid** | **Isopleth area** | **Site at centroid** | **Isopleth area** | **Site at centroid** |
| 3781 | 693.63 | Dog’s household | 6200.88 | Dog’s household | 39026.34 | Next to dog's household |
| 3783 | 522.52 | Dog’s household | 5398.19 | Dog’s household | 967.95 | Bush next to maize field |
|  | 1029.38 | Rubbish dump | 6455.43 | Rubbish dump | 57762.54 | Rubbish dump close to dog’s household |
|  |  |  | 2398.62 | Human compound (house occupant confirmed that she has no dogs but sees other dogs enter her compound) | 1449.71 | Garden in a human compound |
|  |  |  |  |  | 19671.70 | Bush next to a human compound |
|  |  |  |  |  | 11722.09 | Market place close to where vegetables and fish are sold |
| 3785 | 701.59 | Dog’s household | 6548.73 | Dog’s household | 49241.45 | Dog’s household |
| 3787 | 794.17 | Dog’s household | 7309.72 | Dog's household | 52455.72 | Maize field |
| 3789 | 753.57 | Dog’s household | 6801.31 | Dog’s household | 47810.15 | Dog’s household |
| 3791 | 877.30 | Dog’s household | 8352.91 | Dog’s household | 72302.72 | Dog’s household |
| 3793 | 913.74 | Human compound (house occupants say that the neighbours' dog likes to come here often) | 9517.36 | Human compound | 4213.75 | Human compounds far from where we sampled |
|  |  |  | 326.36 | Human compound (of dog 3791) | 32915.99 | Next to a maize field |
|  |  |  |  |  | 62213.93 | Bushy area |
|  |  |  |  |  | 520.20 | Human compound |
|  |  |  |  |  | 375.75 | Rubbish dump outside a human compound |
| 3795 | 0.59 | Human compound | 5817.28 | Human compound | 106028.31 | Maize field |
|  | 1653.09 | Dog’s household | 11164.27 | Dog’s household | 2701.75 | Garden next to a human compound |
| 3797 | 768.19 | Dog’s household | 7892.43 | Dog’s household | 94602.52 | Next to a maize field |
|  |  |  |  |  | 17971.93 | Human compound close to pit latrines |
|  |  |  |  |  | 883.78 | Human compound |
|  |  |  |  |  | 1418.52 | Rubbish dump next to a rental compound |
|  |  |  |  |  | 8910.74 | Human compound |
|  |  |  |  |  | 952.49 | Next to a pit latrine |
|  |  |  |  |  | 2305.55 | Rubbish dump within a rental compound (house occupants confirmed that they see dogs here) |
| 3799 | 1135.03 | Dog’s household | 14025.24 | Dog’s household | 4200.61 | Maize field quite far from dog’s household |
|  |  |  |  |  | 167533.63 | Open space next to a field (human faeces was present) |
|  |  |  |  |  | 2502.05 | Grassy area outside a human compound |
|  |  |  |  |  | 383.46 | By the side of the road next to a field |

6) Malaba

|  | **10%** |  | **50%** |  | **90%** |  |
| --- | --- | --- | --- | --- | --- | --- |
| **ID** | **Isopleth area** | **Site at centroid** | **Isopleth area** | **Site at centroid** | **Isopleth area** | **Site at centroid** |
| 2766 | 722.32 | Dog's household | 6316.78 | Dog's household | 43376.54 | Dog’s household |
|  |  |  |  |  | 1339.52 | Human compound under construction (same site visited by dog 2774) |
| 2770 | 952.40 | Dog's household | 9368.92 | Dog's household | 126837.67 | Holding ground with poultry farm and other dogs |
|  |  |  |  |  | 15810.93 | Field next to a river at the border with Uganda |
|  |  |  |  |  | 7695.56 | Small rubbish dump by the roadside (same site visited by dog 2768) |
|  |  |  |  |  | 3392.35 | Large rubbish dump site across the road (observed dogs at this site) |
| 2768 | 664.83 | Dog's household | 6057.57 | Dog's household | 69257.98 | Human compound (house occupant confirmed that other dogs visit her household at night) |
|  |  |  |  |  | 3000.26 | Small rubbish dump by the roadside (same site visited by dog 2770) |
|  |  |  |  |  | 2940.49 | Common water point (observed some dogs around this area) |
| 2772 | 1525.54 | Dog's household | 14077.28 | Dog's household | 50607.17 | An empty uninhabited building where firewood is stored |
|  |  |  |  |  | 88956.29 | Dog's household |
|  |  |  |  |  | 2808.59 | Water sites in a mosque where people clean themselves before entering the mosque |
| 2774 | 1560.07 | Dog’s household | 3382.10 | Rubbish dump quite far from dog's household | 96157.45 | Human compound under construction (same site visited by dog 2766) |
|  |  |  | 19309.54 | Dog's household | 83966.58 | Dog's household |
|  |  |  |  |  | 4700.51 | Field by the river (observed children chasing monkeys from the crops) |
| 2776 | 1063.84 | Calf pen within dog's household | 12170.08 | Dog's household | 1772.50 | Rubbish dump next to human compounds |
|  | 505.68 | Field next to household of dog 2774 |  |  | 64902.18 | Dog's household |
| 2780 | 1635.74 | Dog's household | 12674.74 | Dog's household | 54700.03 | Field within dog's household |
| 2782 | 675.14 | Dog's household | 7308.72 | Dog's household | 57584.61 | Dog's household |

7) Mudembi

|  | **10%** |  | **50%** |  | **90%** |  |
| --- | --- | --- | --- | --- | --- | --- |
| **ID** | **Isopleth area** | **Site at centroid** | **Isopleth area** | **Site at centroid** | **Isopleth area** | **Site at centroid** |
| 2745 | 1526.22 | Dog's household | 18887.24 | Dog's household | 22861.32 | Maize field with grazing cows |
|  |  |  |  |  | 5741.84 | Sand harvesting point |
|  |  |  |  |  | 1300.54 | Grassy area next to a human compound |
|  |  |  |  |  | 2126.94 | Next to fence of a human compound |
|  |  |  |  |  | 1639.73 | Human compound |
|  |  |  |  |  | 7271.62 | In front of a human compound |
|  |  |  |  |  | 272948.52 | Under a tree in a human compound |
|  |  |  |  |  | 1215.13 | Maize field |
|  |  |  |  |  | 7920.04 | Mango field |
|  |  |  |  |  | 5526.51 | Roadside next to a maize field |
| 2747 | 950.89 | Dog's household | 6325.40 | Dog's household | 86.32 | Human compound far from the dog's household (observed female dogs in the compound) |
|  |  |  | 4001.36 | Next to a butchery | 263.05 | Human compound quite far from the dog's household |
|  |  |  |  |  | 27950.88 | Grassy area within a human compound |
|  |  |  |  |  | 20869.63 | Bushy area in front of human compounds |
|  |  |  |  |  | 800.39 | Grassy area within a human compound |
|  |  |  |  |  | 123541.98 | Rubbish dump next to a small compound where movies are shown |
|  |  |  |  |  | 1238.05 | Field by human compound and next to a shop (observe many dogs sleeping here) |
|  |  |  |  |  | 7654.12 | Bushy area beside a human compound |
| 2749 | 740.43 | Dog's household | 6933.46 | Dog's household | 74754.90 | Tree by the dog's household |
|  |  |  |  |  | 3458.32 | Human compound (house occupant confirmed that she has a dog she observes other dogs visit her compound) |
|  |  |  |  |  | 25661.36 | Outside a human compound |
| 2751 | 579.42 | Dog's household | 5433.37 | Dog's household | 49266.49 | Dog's household |
| 2753 | 1421.12 | Dog's household | 12870.15 | Dog’s household | 78273.24 | Bushy area next to the dog's household |
|  |  |  |  |  | 4630.89 | Grassy area next to human compounds |
| 2758 | 1073.91 | Dog's household | 12492.48 | Grassy area close to rubbish dump | 967.30 | Human compound (observed dogs in the compound) |
|  |  |  |  |  | 71465.56 | Rubbish dump next to pit latrines |
|  |  |  |  |  | 4062.27 | Uninhabited human compound surrounded by fields (a woman working in the fields told us that the owner of the house had dogs, but she died around two years ago and the dogs have since relocated) |
|  |  |  |  |  | 5073.50 | Grassy area next to a water pump |
|  |  |  |  |  | 9220.46 | Field next to river Nzoia (man working the field here mentioned that he often sees dogs come here to chase monkeys) |
| 2760 | 1330.47 | Human compound | 3511.42 | Human compound | 109228.66 | Human compound |
|  |  |  | 12981.26 | Human compound | 506.88 | Maize and millet field |
|  |  |  |  |  | 7260.13 | Human compound (household of dog 2747) |
|  |  |  |  |  | 5462.89 | Rubbish dump in a culvert by the road |
| 2762 | 933.43 | Dog's household | 12031.62 | Dog's household | 89633.50 | Grassy area close to dog’s household |
| 2764 | 447.95 | Dog's household | 3465.47 | Dog's household | 19592.61 | Dog's household |

8) Nambale

|  | **10%** |  | **50%** |  | **90%** |  |
| --- | --- | --- | --- | --- | --- | --- |
| **ID** | **Isopleth area** | **Site at centroid** | **Isopleth area** | **Site at Centroid** | **Isopleth area** | **Site at centroid** |
| 3753 | 988.26 | Dog’s household | 8248.60 | Dog’s household | 10328.51 | Rubbish dump between human compounds |
|  |  |  |  |  | 64402.72 | Sweet potato field |
|  |  |  |  |  | 18868.14 | Rubbish dump next to a human compound (same site visited by dog 2795) |
|  |  |  |  |  | 1661.98 | Rubbish dump next to a hotel |
|  |  |  |  |  | 2946.42 | Maize field |
| 3756 | 213.61 | Dog's household | 5514.16 | Dog's household | 42597.45 | Human compound (household of dog 2790) |
|  |  |  |  |  | 2338.87 | Rubbish dump next to rental compounds |
|  |  |  |  |  | 34557.14 | Mound in a maize field (observed many dogs at this site) |
|  |  |  |  |  | 1112.09 | Grassy area by the river |
| 2795 | 754.92 | Dog's household | 7070.75 | Dog's household | 45065.93 | Dog's household |
|  |  |  |  |  | 26159.38 | Rubbish dump next to a human compound (same site visited by dog 3753) |
|  |  |  |  |  | 518.92 | Rubbish dump next to a rental houses |
|  |  |  |  |  | 595.69 | Human compound (household of dog 3760) |
| 3758 | 1964.24 | Maize fields next to river | 682.14 | Big rubbish dump within bus station (same site visited by dog 2788) | 135064.32 | Rental houses with no fences |
|  |  |  | 8332.45 | Bushy area next to human compounds (observed pigs tethered here) | 1557.90 | Rubbish dump just outside the dog's household |
|  |  |  | 14047.33 | Maize field next to river | 3276.47 | Grassy area by the river |
| 3760 | 614.24 | Maize fields | 1206.0 | Dog's household  (owners confirmed that the dog is usually kept in the field to protect crops from monkeys) | 23722.78 | Dog's household |
|  |  |  | 5724.32 | Maize fields | 33811.03 | Maize fields |
| 2788 | 504.89 | Dog's household | 3538.21 | Big rubbish dump within bus station (same site visited by dog 3758) | 68247.12 | Rubbish dump just outside the dog's household |
|  | 1193.01 | Maize fields | 7839.66 | Dog's household | 2729.09 | Maize field |
|  |  |  | 7398.96 | Maize fields | 11474.35 | Behind the slaughterhouse (observed slaughterhouse workers throw left-overs in this site) |
|  |  |  |  |  | 47123.49 | Maize fields |
|  |  |  |  |  | 3745.52 | Grassy area by the river |
| 2786 | 1216.33 | Dog's household | 11986.80 | Dog's household | 31217.93 | Field close to rental houses |
|  |  |  |  |  | 1095.01 | Maize fields |
|  |  |  |  |  | 106556.39 | Field next to dog's household |
|  |  |  |  |  | 7747.09 | Rubbish dump behind a supermarket |
|  |  |  |  |  | 19484.64 | Bushy area opposite the slaughterhouse |
| 2790 | 1169.46 | Dog's household | 11114.09 | Dog's household | 70979.24 | Dog's household |
| 4288 | 1187.60 | Next to a human compound (house occupants confirmed that they had no dogs) | 5204.19 | Rubbish dump by the roadside | 126010.49 | Rubbish dump with a human compound |
|  |  |  | 14911.70 | Next to a human compound | 10264.68 | Within slaughterhouse compound |
